# Untargeted Metabolomic Profiling Reveals a Distinct Intestinal Metabolic Signature in Necrotizing Enterocolitis

**DOI:** 10.3390/microorganisms14102202

**Published:** 2026-10-01

**Authors:** Joann Romano-Keeler, Qianming Jiang, De-Ann Pillers, Muthusamy Thiruppathi, Hua Geng, Thom E. Lobe, Xiao-Di Tan, Joern-Hendrik Weitkamp

**Affiliations:** 1 Department of Pediatrics, University of Illinois Chicago, Chicago, IL 60607, USA; qj5@uic.edu (Q.J.); pillersd@uic.edu (D.-A.P.); tmrarl77@gmail.com (M.T.); hgeng5@uic.edu (H.G.); xtan25@uic.edu (X.-D.T.); 2 Department of Surgery, Division of Pediatric Surgery, University of Illinois Chicago, Chicago, IL 60607, USA; tlobe@uic.edu; 3 Department of Pediatrics, Vanderbilt University Medical Center, Nashville, TN 37232, USA; hendrik.weitkamp@vumc.org

**Keywords:** metabolomics, necrotizing enterocolitis, intestinal tissue, metabolites, newborn, prematurity

## Abstract

Necrotizing enterocolitis (NEC) is a devastating gastrointestinal emergency affecting up to 10% of infants < 1500 g. Although prior metabolomic studies have profiled metabolite derangements, few have focused on intestinal tissue, the primary site of injury. In this study, we performed metabolomic profiling of intestinal tissue from patients with NEC and surgical controls to identify metabolic alterations associated with NEC. Tissue was collected during surgery from twenty-one infants (8 controls, 13 NEC). Samples underwent liquid chromatography–tandem mass spectrometry. Raw data were extracted, peak-identified, and quality-control-processed, and compounds were identified by comparison with a purified standards library. Following normalization and log transformation, a mixed-effects model accounting for repeated samples from individual infants was used to identify metabolites that differed between NEC and control tissue. There were no significant differences in sex, gestational age, days of life, antibiotic use, or type of feeds between groups. Principal component analysis and hierarchical clustering demonstrated partial separation of NEC and control samples. Metabolomic profiling identified 1183 biochemical features, with 308 demonstrating nominal differential abundance between NEC and control tissue. Indole-related metabolites were increased in NEC tissue, including the nominally significant 3-indoxyl sulfate, a host–microbial co-metabolite associated with epithelial barrier dysfunction. Caffeine-related metabolites were also increased in NEC tissue, while docosahexaenoate demonstrated a trend toward increased relative abundance that did not reach statistical significance. Enrichment of retinol metabolism was observed in NEC tissue, suggesting alterations in retinoid-associated metabolic pathways. Hydroxybutyrate demonstrated a nonsignificant trend toward decreased abundance in NEC. Overall, NEC intestinal tissue demonstrated broad metabolic alterations compared with surgical controls, including differences in lipid, retinol, and host–microbial metabolic pathways. These findings characterize the intestinal metabolic environment associated with NEC and highlight the value of tissue-based metabolomics for understanding disease-associated intestinal alterations.

## 1. Introduction

Necrotizing enterocolitis (NEC) is a life-threatening gastrointestinal disease affecting approximately 5–10% of preterm infants with a birth weight under 1500 g [1,2,3]. Its pathogenesis is multifactorial, involving prematurity, infectious triggers, immune dysregulation, hypoxic–ischemic injury, and immaturity of the intestinal barrier [4]. Approximately 40% of affected infants require surgical resections often resulting in lifelong complications, including short gut syndrome, malabsorption, and impaired growth [5]. The mortality rate of preterm infants with NEC varies between 20 and 30%, reaching up to 50% mortality in extremely low-birth-weight infants (<1000 g) [6].

Currently, the diagnosis of NEC is made by radiographic findings, clinical examination, and laboratory studies. However, many of these items can be non-specific and challenging to interpret such that patients with sepsis or other infectious entities can often be misdiagnosed with NEC. The evolution of “-omics” technologies over the last decade, including the microbiome, metabolome, and proteome, have generated an interest in defining signature microbes, metabolites, or proteins in NEC patients [7,8]. Such signatures could inform our understanding of risk factors for NEC or serve as markers for predicting NEC in high-risk patients.

Metabolomics is a high-throughput analytical approach that examines the full spectrum of small-molecule metabolites (<2 kDa) within a biological system at a specific time, utilizing bodily fluids such as urine, blood, and stool, enabling real-time profiling of metabolic phenotypes [9,10]. Unlike genomic or transcriptomic markers, metabolites are the final products of gene transcription and enzymatic pathways, allowing them to accurately reflect an organism’s phenotype and biological activity. However, metabolite composition is influenced by various factors, including environmental interactions, diet, and exposure to toxins or medications, which collectively shape the true phenotype. This complex interplay between metabolites and external factors highlights the intricacy of analysis and interpretation of data generated from metabolomic studies.

Several metabolomic studies in NEC have been conducted using urine, stool, and plasma samples. A recent systematic review of five untargeted studies did not identify a unifying NEC-specific signature but revealed consistent disruptions in pathways linked to inflammation, intestinal permeability, and energy metabolism [11,12,13,14,15]. Notably, none of these five studies employed intestinal tissue. Prospective urine-based analyses have reported distinct metabolic profiles between medical and surgical NEC, including elevated N-acetylaspartic acid, butyrylcarnitine, and propionylcarnitine in medical NEC [16]. Other work suggests that urinary metabolites such as lactate, betaine, myo-inositol, urea, creatinine, and N,N-dimethylglycine may help differentiate NEC from food intolerance [17]. Fecal metabolomics has further demonstrated dynamic early-life changes in preterm infants, with altered amino acid metabolism and energy depletion pathways in those who later develop NEC [18].

However, despite the site of injury being at the intestinal mucosa and the clinical significance of metabolites produced at the affected area, metabolomic studies in NEC tissue are sparse. Data from other sources must be extrapolated and interpreted for tissue level processes. In this study, we provide tissue level metabolomics data from premature infants with NEC and control infants requiring intestinal resections for non-NEC indications. We detected distinct metabolite profiles separating patients with NEC from age and gestational age matched non-NEC surgical control patients leading to new hypotheses on the pathogenesis of NEC.

## 2. Materials and Methods

### 2.1. Subject Recruitment and Sample Retrieval

This study was approved by the Institutional Review Board at University of Illinois Chicago (UIC) and Vanderbilt University. All infants hospitalized in the neonatal intensive care unit at UIC and at the Monroe Carrell Jr. Children’s Hospital at Vanderbilt were eligible for this study if they were <180 days of age and required intestinal surgery due to NEC or other etiologies. Patients having intestinal resections due to etiologies other than NEC were incorporated into the control group. We obtained written informed consent from parents, the next of kin, caretakers, or guardians on behalf of the minors/children enrolled in the study to permit collection of tissues. Infants were excluded if consent could not be obtained. Consent for this study was obtained on a single occasion. If patients underwent multiple surgeries involving intestinal tissue resection, additional consent was not required for tissue collection at subsequent surgeries, consistent with the institutional IRB-approved protocols. A subset of infants included in the present cohort was included in previously published studies from this biorepository; however, the analyses and outcomes reported here are distinct and have not been previously published [8,18]. Intestinal tissue samples were collected sterilely at the time of surgery and while samples were identified as either jejunum, ileum, duodenum, or large intestine, more specific information on the anatomic location was not always available. Samples were immediately snap frozen and placed directly in −80 °C or placed directly in −80 °C. Freezing time was variable and was based on the date of collection relative to the date samples were processed. To eliminate batch effects, all samples were processed for metabolomics studies in the same batch and on the same day. An approximate sample weight of 24 mg was used for each patient to achieve adequate sample volume for metabolomics.

### 2.2. Demographic and Clinical Data Collection

Demographic data were collected at the time of enrollment and included (1) weight; (2) gestational age; (3) sex; (4) mode of delivery; (5) enteral feeding regimen; (6) type of surgical resection; (7) mode of delivery; and (8) antibiotic exposure. This information was prospectively entered into a HIPAA compliant database which was only accessible to IRB trained study personnel.

### 2.3. Tissue Sample Preparation

Tissue sample preparation and global untargeted metabolomic analysis were performed by Metabolon (Metabolon Inc., Morrisville, NC, USA). Briefly, thawed samples were precipitated with methanol under vigorous shaking for two minutes with a Genogrinder 2000 to remove protein and recover chemically diverse metabolites (Glen Mills, Clifton, NJ, USA). The resulting supernatant was divided into multiple fractions for analysis with UPLC-MS/MS (positive mode), UPLC-MS/MS (negative mode), and hydrophilic interaction liquid chromatography (HILIC) UPLC-MS/MS. Samples were briefly placed on a TurboVap^®^ (Zymark, Hopkinton, MA, USA) to remove organic solvent and then stored overnight under nitrogen to prevent oxidation before analysis.

To assess technical variability, technical replicates were generated by pooling small aliquots of experimental samples and injected periodically throughout the platform run. Extracted water samples were included as process blanks to detect background contamination. A combination of quality control (QC) standards, carefully selected to avoid interference with endogenous metabolite measurements, was added. Experimental samples were randomized across the platform run to minimize batch effects, with QC samples spaced evenly among the injections to ensure robust data quality monitoring.

### 2.4. Ultrahigh Performance Liquid Chromatography–Tandem Mass Spectrometry (UPLC-MS/MS)

All methods used an ACQUITY UPLC system (Waters, Milford, MA, USA) coupled to a Q-Exactive mass spectrometer (Thermo Fisher, Waltham, MA, USA) with a heated electrospray ionization (HESI-II) probe (Thermo Fisher, Waltham, MA, USA) and an Orbitrap mass analyzer (Thermo Fisher, Waltham, MA, USA) at 35,000 mass resolution. Dried sample extracts were reconstituted in method-specific solvents containing internal standards for injection and chromatographic consistency. Four analytical methods were used:

1. PosEarly (Hydrophilic Compounds, Positive Ion Mode): Gradient elution from a C18 column (2.1 × 100 mm, 1.7 µm) with water/methanol containing 0.05% perfluoropentanoic acid (PFPA) and 0.1% formic acid (FA).
2. PosLate (Hydrophobic Compounds, Positive Ion Mode): Similar C18 column with methanol, acetonitrile, water, 0.05% PFPA, and 0.01% FA, optimized for higher organic content.
3. Neg (Basic Negative Ion Mode): Separate C18 column with methanol/water and 6.5 mM ammonium bicarbonate (pH 8).
4. HILIC (Negative Ion Mode): HILIC column (2.1 × 150 mm, 1.7 µm) with water/acetonitrile and 10 mM ammonium formate (pH 10.8).

MS analysis alternated between MS and data-dependent MSn scans with dynamic exclusion, covering 70–1000 *m*/*z*.

### 2.5. Data Extractions and Compound Identification

Untargeted metabolomic profiling was performed using Metabolon’s global metabolomics platform, in which LC-MS/MS data were acquired without restricting analysis to a predefined panel of metabolites. Raw metabolomic data were extracted, peak-identified, and quality-controlled using proprietary software developed by Metabolon, Inc. (Morrisville, NC, USA) Compounds were assigned by comparison to Metabolon’s reference library of purified standards and recurrent unknown entities, which includes retention indices (RI), mass-to-charge ratios (*m*/*z*), and fragmentation spectra. Identification was based on three criteria: (1) RI within a narrow window of the proposed compound, (2) accurate mass match to the library (±10 ppm), and (3) forward and reverse MS/MS spectral scores comparing experimental data with authentic standards. Applying all three parameters ensured reliable discrimination among structurally similar molecules.

The library contains >5400 purified standards characterized across all analytical platforms, along with ~7000 recurrent but structurally unnamed compounds catalogued by consistent chromatographic and mass spectral features. These unnamed biochemicals may be fully annotated as matching standards are acquired or through structural analysis. The library is continuously expanded with biologically relevant compounds to improve the scope and accuracy of metabolite identifications.

### 2.6. Statistical Analyses

Initial metabolite processing and normalization were performed by Metabolon, Inc. Processed metabolomics data were subsequently analyzed using MetaboAnalyst 6.0 (https://www.metaboanalyst.ca, accessed on 28 July 2026) for heat maps, pathway enrichment analysis, principal component analysis (PCA), and hierarchical clustering analysis (HCA). Following normalization to the sample mass extracted, metabolite values were scaled to the median value and log2-transformed prior to statistical analysis. Missing metabolite measurements were excluded on a metabolite-specific basis, and each model was fit using all available observations for that metabolite. For the primary analysis, a mixed-effects model was used to identify metabolites that differed between NEC and control samples, with disease group included as a fixed effect and patient included as a random effect to account for repeated samples from the same individual. Fold changes and the corresponding 95% confidence intervals were derived from the estimated group effect and expressed as NEC relative to control. Benjamini–Hochberg false discovery rate (FDR) correction was applied across all metabolites with estimable mixed-effects model *p*-values (n = 998).

As a secondary analysis to evaluate the potential influence of control type, samples were stratified into three groups: active NEC (n = 13), non-ostomy surgical controls (n = 3), and ostomy takedown/reanastomosis controls (n = 5). Metabolite abundance was compared among these groups using linear regression with surgical group as a categorical predictor and patient-clustered standard errors to account for the three infants who contributed samples during both active NEC and subsequent ostomy takedown. Overall differences among the three groups were evaluated, followed by pairwise comparisons between active NEC and non-reanastomosis surgical controls, active NEC and reanastomosis controls, and non-ostomy surgical controls and reanastomosis controls.

Multiple testing was addressed using the Benjamini–Hochberg false discovery rate (FDR) procedure, and FDR-adjusted *q*-values were calculated. Pathway enrichment analysis was performed to identify metabolic pathways significantly overrepresented among metabolites showing differential abundance between groups. Metabolite identities were annotated using KEGG and pathways with adjusted *p*-values < 0.05 were considered significantly enriched. Unsupervised multivariate analyses, including principal component analysis (PCA) and hierarchical clustering analysis (HCA; Euclidean distance, Ward’s linkage), were performed to evaluate global metabolic patterns.

## 3. Results

### 3.1. Clinical Characteristics of the Cohort

We collected and analyzed fresh surgical tissue from 13 patients with NEC and 8 patients without NEC; in total, 21 samples were analyzed. Surgical samples from patients without NEC included infants with spontaneous intestinal perforation (SIP) (n = 2), midgut volvulus (n = 1), and ostomy takedown and reanastomosis after either NEC (n = 4) or SIP (n = 1). Any patient with surgical NEC was classified as Bell’s Stage III. All patients had segmental bowel resections to address injured or necrotic intestinal segments and in several cases, ostomies were created. Among the five patients who had ostomies, two were ileostomies, two were jejunostomies, and one was a colostomy. Surgical approaches to SIP or other anatomic variants (midgut volvulus) were variable. Except for two colon samples within the NEC group and one colon sample within the control group, all tissues analyzed were either jejunum or ileum. Mean gestational age, birth weight, and postnatal age were 28 weeks (range 23–33 weeks), 957 g (range 500–1740 g), and 24 days (range 6–60 days) for NEC patients and 29 weeks (range 23–39 weeks), 1177 g (range 500- 2590 g), and 51 days (range 1–105 days) for control patients (Table 1). There were no statistically significant differences in any of these metrics between groups (*p* > 0.05). Both the NEC and non-NEC groups included infants receiving breast milk, formula, or no enteral nutrition. Males represented 62% of both the NEC and non-NEC groups. Delivery mode was via Caesarean section in 62% (n = 5) of control patients and 61% (n = 8) of NEC patients. All patients in the control and NEC groups received early antibiotics. Approximately 62% of control patients and 54% of NEC patients received antibiotics >24 h prior to surgery, which was not a statistically significant difference. Three patients were represented in both the control and NEC groups since they had tissue resected during their initial NEC surgery (NEC group) and then tissue resected 6–8 weeks after infection had resolved during ostomy takedown and intestinal reanastomosis (Control group).

### 3.2. Separation of Groups by Hierarchical Clustering and Principal Component Analysis

Untargeted metabolomic profiling detected 1183 biochemicals in intestinal tissue, including 1039 annotated metabolites and 144 structured unnamed biochemicals. Using a mixed-effects model to account for repeated samples from individual infants, 308 metabolites were differentially abundant between NEC and control tissues at a nominal significance threshold of *p* < 0.05, representing 26% of all detected biochemicals. Of these, 241 metabolites were increased and 67 were decreased in NEC relative to control tissues. Following Benjamini–Hochberg false discovery rate (FDR) correction across metabolites with estimable mixed-effects model *p*-values, 236 metabolites remained significant at *q* < 0.10, including 191 metabolites at *q* < 0.05. Of the 308 differentially abundant metabolites, 255 were annotated as known metabolites and are reported with their nominal *p*-values and FDR-adjusted q-values in Supplementary Table S1; unidentified metabolites are excluded from the table.

**Figure 1:**
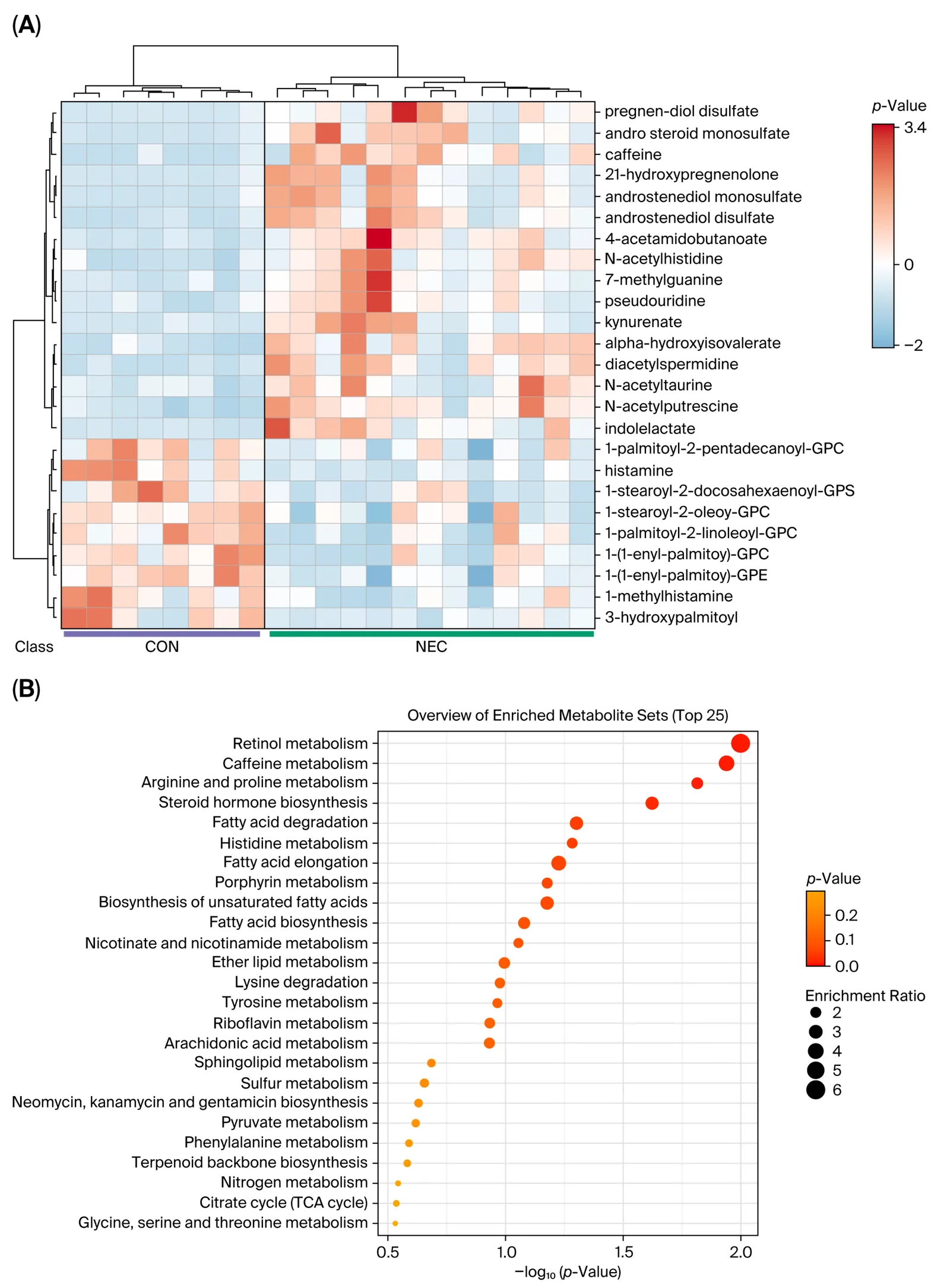
Heat map and enrichment analysis of control and NEC tissue samples. (**A**) Hierarchical clustering of the top 25 differentially abundant metabolites for control and NEC group samples. Experimental group is along the *x*-axis with NEC samples in green and control samples in purple. (**B**) Quantitative enrichment analysis of top 25 enriched metabolites across control and NEC intestinal tissue samples.

Hierarchical clustering heat map analysis using Ward’s method of the top 25 differentially abundant metabolites demonstrated separation of NEC (green) versus control (purple) patients based on intestinal tissue metabolite profiles (Figure 1A). Pathway enrichment analysis was then performed and the top 25 enriched and biologically meaningful pathways are shown in Figure 1B. Among these, retinol metabolism (*p* = 0.010), caffeine metabolism (*p* = 0.010), arginine and proline metabolism (*p* = 0.015), steroid hormone biosynthesis (*p* = 0.023), and fatty acid degradation (*p* = 0.049) were the most significantly enriched pathways distinguishing NEC from control tissue.

**Figure 2:**
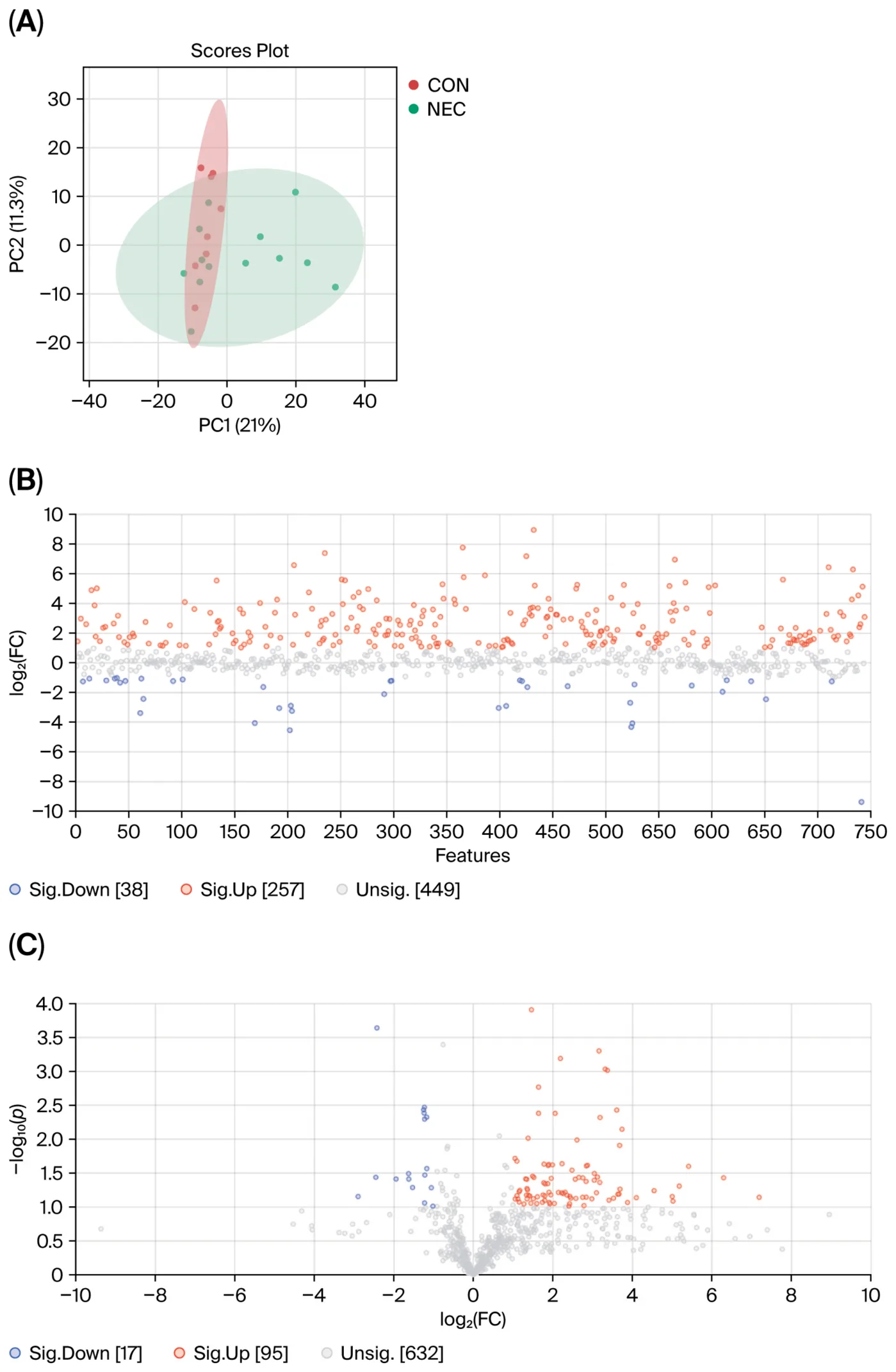
Principal Component Analysis (PCA) and fold change analysis of intestinal tissue from Control and NEC groups. (**A**) PCA score plot showing partial separation between NEC (red) and control (green) intestinal tissue samples based on normalized metabolite concentrations. Ellipses represent the 95% confidence intervals for each group. (**B**) Fold-change analysis comparing mean metabolite concentrations between NEC and control samples using a twofold threshold. Metabolites elevated in NEC are shown in red and those decreased in blue. (**C**) Volcano plot integrating fold-change threshold of 2.0 and statistical significance with *p* < 0.05. Metabolites significantly upregulated in NEC are shown in red (n = 95), those significantly downregulated in blue (n = 17), and nonsignificant metabolites in gray, illustrating a metabolic profile associated with NEC.

Principal component analysis (PCA), which was performed on normalized metabolite concentrations, demonstrates partial separation of the biochemical profiles of NEC (red) versus control (green) patients with PC1 and PC2 accounting for 21% and 11.3% variability, respectively (Figure 2A). NEC patients exhibited greater dispersion, suggesting increased metabolic heterogeneity, whereas controls clustered more tightly, indicating more uniform metabolic profiles. Fold-change analysis comparing mean metabolite concentrations between groups, using a twofold threshold, identified approximately 257 metabolites that were increased and 38 that were decreased in NEC tissue relative to controls (Figure 2B). Volcano plot analysis integrating both > 2-fold-change threshold and nominal *p* < 0.05 identified 95 metabolites with increased abundance and 17 with decreased abundance in NEC tissue (Figure 2C), highlighting a distinct metabolic profile associated with NEC.

### 3.3. Differences in Amino Acid Metabolism Observed Between NEC and Control Patients

**Figure 3:**
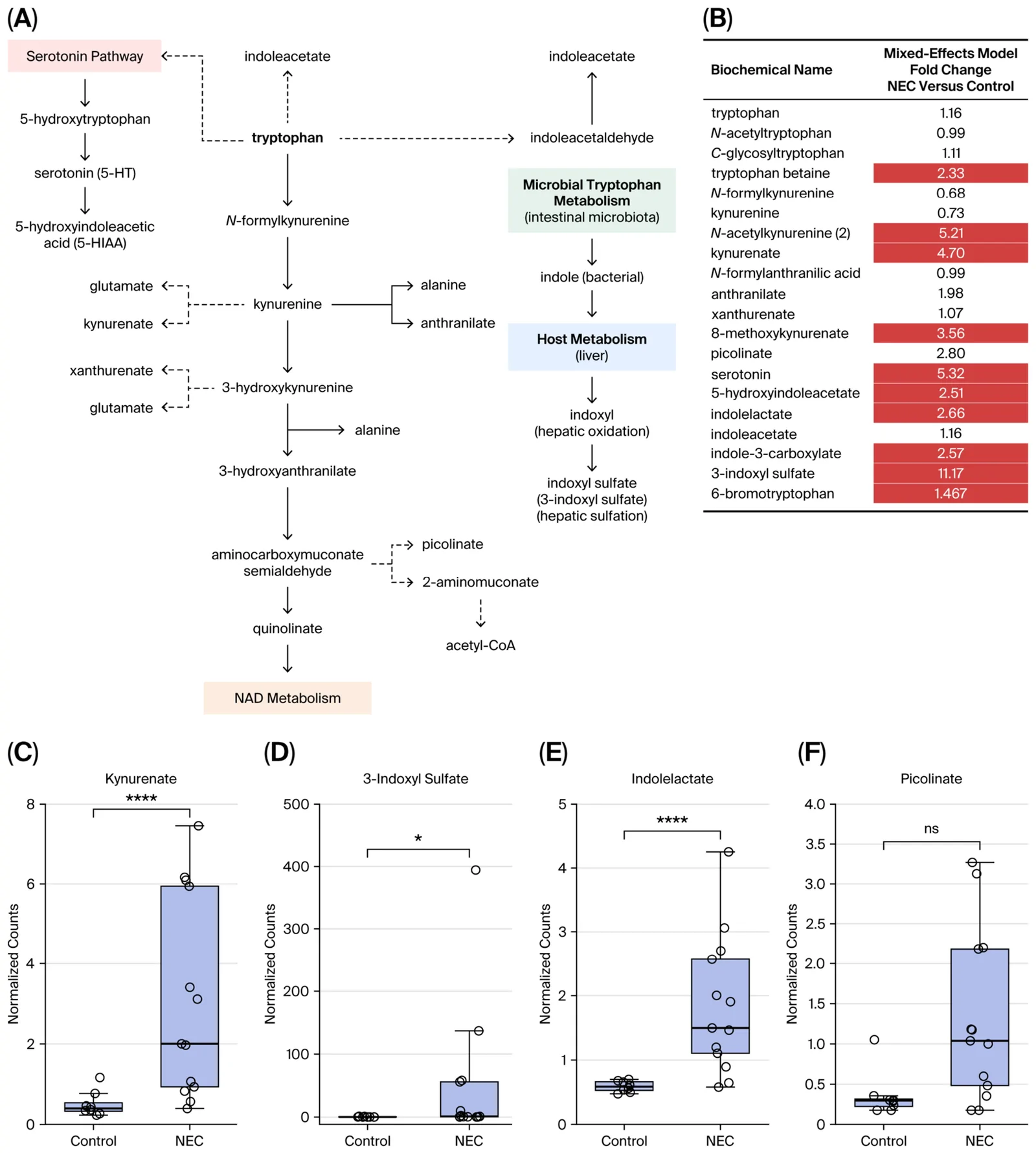
Pathway diagram (**A**), statistical heatmap (**B**), and boxplots (**C**–**F**) of select metabolites associated with tryptophan metabolism. Within the heatmap, trending (0.05 < *p* < 0.10) and significant (*p* ≤ 0.05) differences are indicated for elevated (red) and decreased changes. For box plots, * = *p* < 0.05; **** = *p* < 0.0001; ns = *p* ≥ 0.05.

Increased activity within the tryptophan metabolism pathway was detected in NEC patients, including statistically significant elevations in kynurenate and indole-containing compounds such as indolelactate and indoleacetate (Figure 3). Meanwhile, NEC patients trended toward a decrease in kynurenine; however, it was not statistically significant. The largest difference observed between NEC and control patients was an 11-fold increase in 3-indoxyl sulfate in NEC patients. However, distribution analysis demonstrated substantial inter-individual variability within the NEC group, suggesting that the increase may have been driven by a subset of NEC samples in the setting of low baseline abundance in controls.

**Figure 4:**
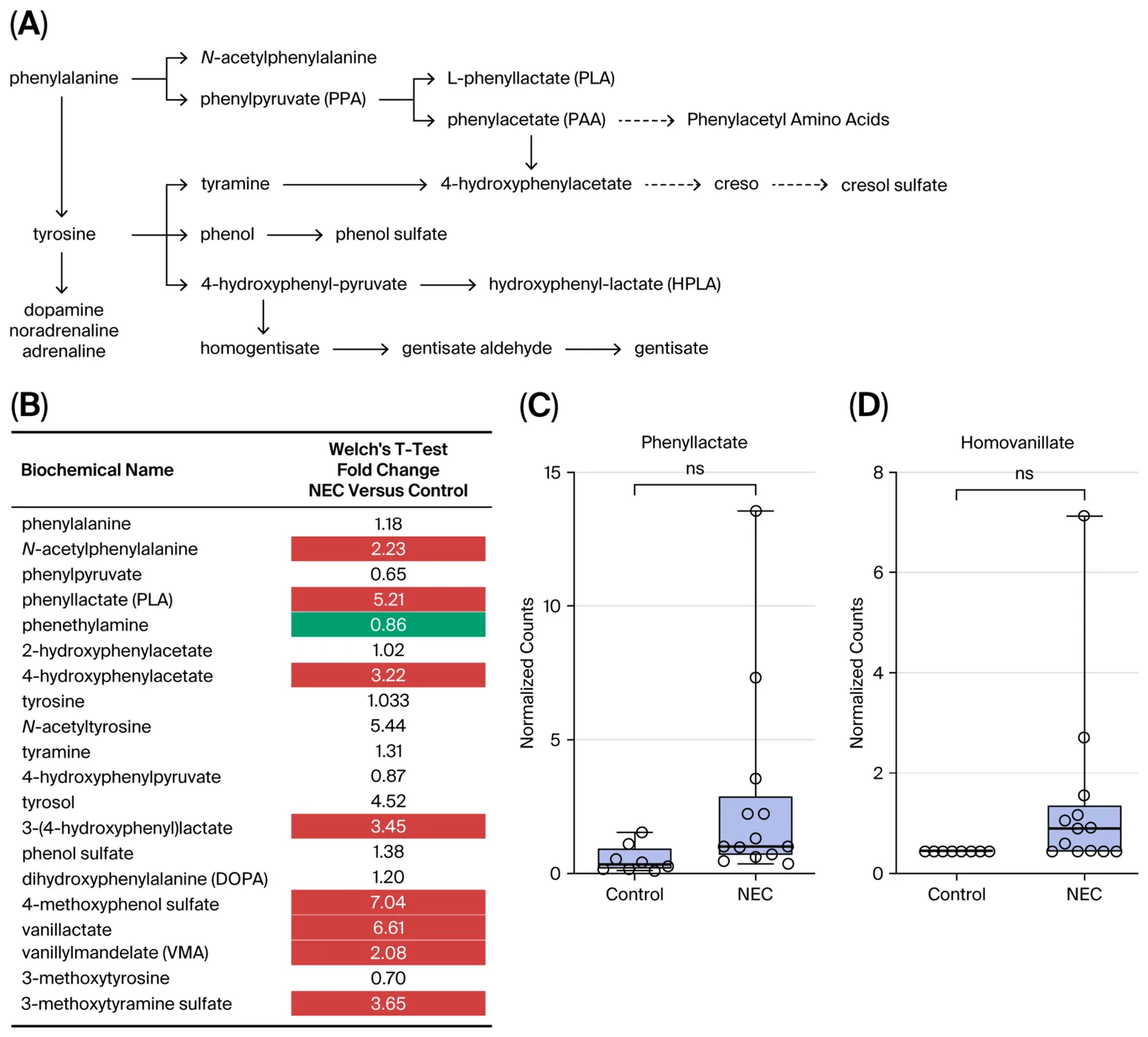
Metabolic pathway schematic (**A**) with accompanying heatmap (**B**) and boxplots (**C**–**D**) demonstrating alterations in tyrosine and phenylalanine metabolism. Within the heatmap, trending (0.05 < *p* < 0.10) and significant (*p* ≤ 0.05) differences are indicated for elevated (red) and decreased (green) changes. For box plots, ns = *p* ≥ 0.05.

The phenylalanine and tyrosine pathways demonstrated multiple metabolites with increased abundance in the NEC group, including a 5-fold increase in 4-hydroxyphenylacetate. Aromatic compounds within the phenylalanine pathway, including N-acetylphenylalanine, phenyllactate (PLA), and 4-hydroxyphenylacetate, were elevated in NEC patients and reached statistical significance. Similarly, metabolites within the tyrosine pathway, including 3-(4-hydroxyphenyl)lactate, 4-methoxyphenol sulfate, and vanillactate and vanillylmandelate (VMA) demonstrated increased abundance in NEC patients compared with controls (Figure 4). Statistically significant elevations in several sulfated derivatives, including 3-methoxytyramine sulfate, homovanillate sulfate, tyramine-O-sulfate, and vanillic alcohol sulfate, were also observed in the NEC group.

**Figure 5:**
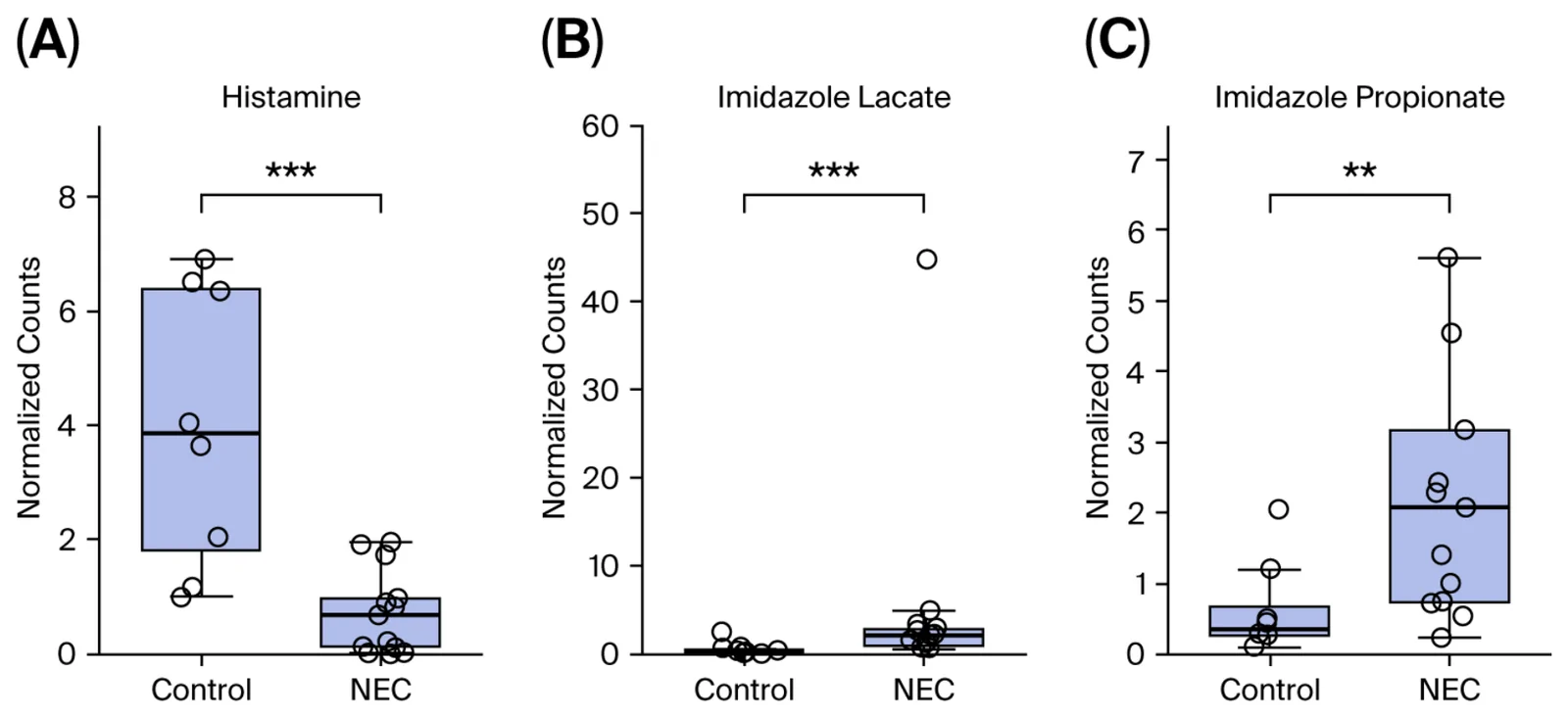
Metabolites of histidine metabolism. Box plots demonstrating alterations in (**A**) histamine, (**B**) imidazole lactate, and (**C**) imidazole propionate in NEC versus controls. ** = *p* < 0.01; *** = *p* < 0.001.

Several metabolites involved in histidine metabolism also differed between NEC and control patients (Figure 5). Histamine, a mediator implicated in intestinal inflammation and gut immune responses, was significantly decreased in NEC patients compared with controls. In contrast, other aromatic metabolites, including imidazole lactate and imidazole propionate, were elevated in the NEC group. To determine whether the single high imidazole lactate value in the NEC group was driving the observed difference, we performed a sensitivity analysis excluding this sample. The difference between NEC and control tissue remained highly significant after exclusion of this observation (*p* = 0.00018), compared with *p* = 0.00020 when all samples were included.

### 3.4. Disturbances in Polyamine and Lysine Metabolism Observed

Several polyamine and associated metabolites were elevated in the NEC group, including those typically associated with bacterial overgrowth, such as cadaverine and N-acetylcadaverine. Although detected in only 60% of NEC samples and 70% of control tissues, cadaverine—a product of lysine metabolism—showed a 25-fold increase in NEC tissue compared to controls. N-acetylcadaverine was detected in all samples but the increase was only 9% in NEC patients versus controls. Putrescine, N-acetylputrescine, N-acetyl-isoputreanine, (N(1) + N(8))-acetylspermidine, N(1)-acetylspermine, N1,N12-diacetylspermine, 5-methylthioadenosine (MTA), and 4-acetamidobutanoate were all elevated in the NEC group. MTA has anti-inflammatory properties, and its elevated signal intensity at the site of injury suggests an immune response to local inflammation secondary to NEC. Minimal changes were observed in urea cycle intermediates (i.e., arginine, ornithine, citrulline) between control and NEC samples.

### 3.5. Increased Detection of Arachidate and Palmitate in NEC Patients

**Figure 6:**
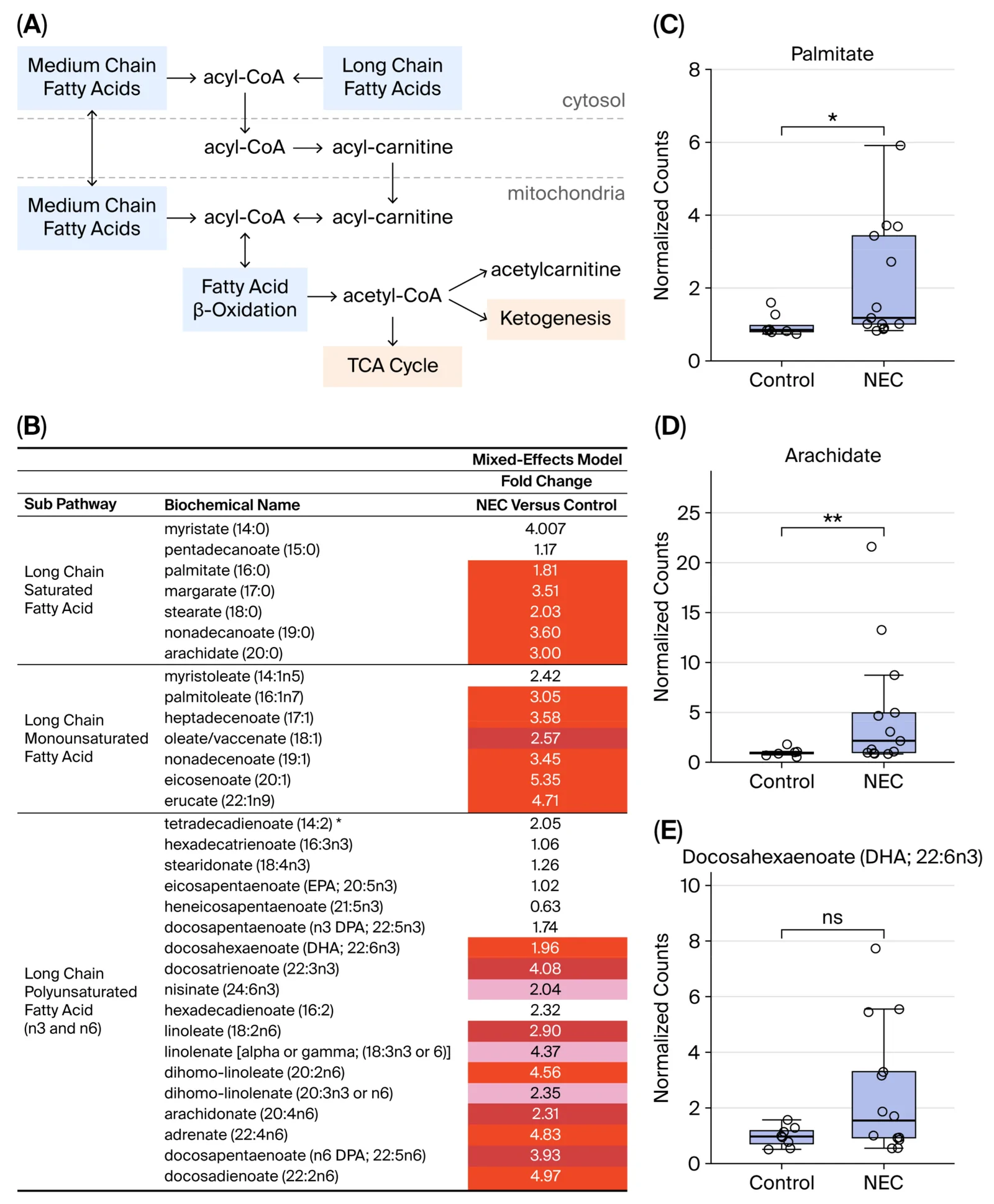
Pathway schematic (**A**), integrated heatmap (**B**), and boxplots (**C**–**E**) showing metabolites associated with fatty acid metabolic pathways. Within the heatmap, trending (0.05 < *p* < 0.10) and significant (*p* ≤ 0.05) differences are indicated for elevated (red/pink) changes. For box plots, * = *p* < 0.05; ** = *p* < 0.01; ns = *p* ≥ 0.05.

Multiple differences in fatty acid metabolites were observed between NEC and control patients (Figure 6). Most long-chain saturated and monounsaturated fatty acids were significantly elevated in NEC patients, including palmitate, arachidate, and eicosenoate. Plasmalogens, the phospholipid species (phosphatidylethanolamines, phosphatidylcholines, and phosphatidylserines), and a few individual acylcarnitines were lower in NEC patients, while others (free fatty acids, endocannabinoids, lysophospholipids, lysoplasmalogens, monoacylglycerols (MAG), and diacylglycerols (DAG)) showed higher abundance, with many individual metabolites remaining significant after FDR correction. The storage lipids (MAG, DAG) may be reporting on absorption differences in the two groups but could also indicate differences in lipase activity and overall lipid metabolism. For the free fatty acids, it is primarily the long-chain species that become elevated. The acylcarnitines (linoleoylcarnitine (C18:2) is shown in Figure 6) as a class show only a few metabolites that are lower while most remain unchanged. Additionally, the ketone 3-hydroxybutyrate (BHBA) was decreased in the NEC group but does not reach statistical significance. Combined, the findings could suggest either decreased β-oxidation in the NEC group, or, alternatively, that the changes in FFA are related to dietary or absorption changes (similar to MAGs and DAGs). The overall profile of lipids (glycerolipids, phospholipids) could be affected by such differences, given the up/down nature of the changes compared to controls.

### 3.6. Methylxanthine Accumulation Noted in NEC Patients

Xanthine metabolites, including caffeine, were detected at statistically significant higher levels in NEC versus control patients. Caffeine and 1,3 dimethylurate had 10-fold higher and 16-fold higher relative abundance, respectively.

### 3.7. Elevations in Progestin Steroid Metabolites in NEC Patients

Metabolites of all classes of steroids, including pregnenolone, progestin, corticosteroids, and androgenic steroids, were elevated in patients with NEC. Specifically, the progestin steroid metabolite, 5α-pregnan-3β, 20α-diol disulfate, was detected almost 22-fold higher in NEC patients.

### 3.8. Secondary Analysis Demonstrates Heterogeneity Among Surgical Controls

**Figure 7:**
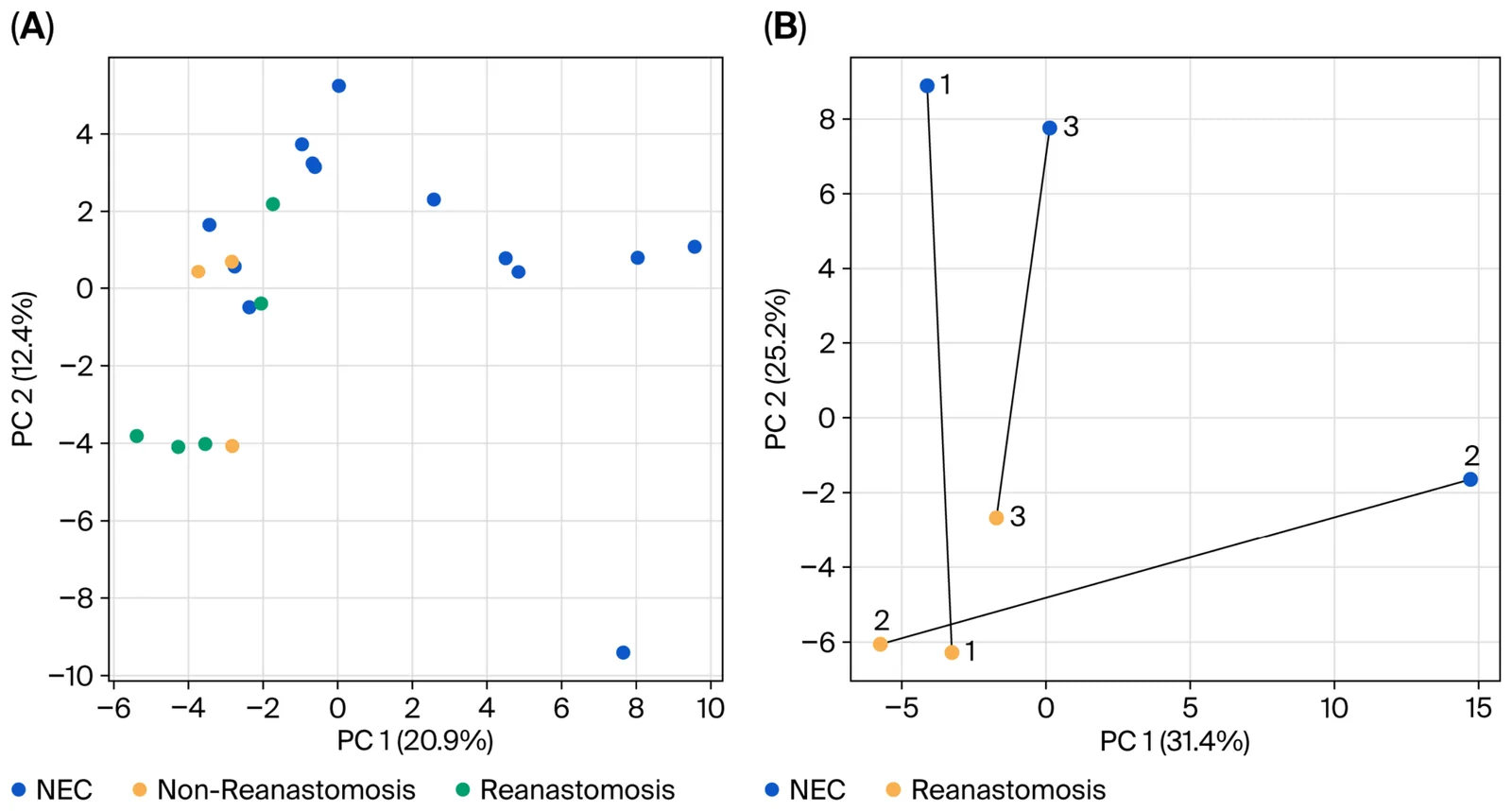
Secondary analyses of surgical controls (**A**) and paired samples (**B**). (**A**) Intestinal metabolomic profiles from infants with active NEC (n = 13) were compared with non-reanastomosis surgical controls obtained during other intestinal surgical procedures (n = 3) and with surgical controls obtained during ostomy takedown/reanastomosis (n = 5). (**B**) Exploratory within-patient analysis of three infants with intestinal tissue collected during active NEC and again at least 6 weeks later during ostomy takedown/reanastomosis.

To evaluate the influence of surgical context on the intestinal metabolomic profile, we performed secondary analyses stratifying control samples according to whether they were obtained during ostomy takedown/reanastomosis or during other surgical procedures. PCA of active NEC (n = 13), non-reanastomosis surgical controls (n = 3), and reanastomosis controls (n = 5) demonstrated that PC1 and PC2 accounted for 20.9% and 12.4% of the variation, respectively (Figure 7A). Differences in the distribution of reanastomosis and non-reanastomosis surgical control samples were observed as consistent with biological heterogeneity within the surgical control group. We also performed an exploratory within-patient analysis of three infants with intestinal tissue collected during active NEC and again at least six weeks later during ostomy takedown/reanastomosis. In this PCA, PC1 and PC2 accounted for 31.4% and 25.2% of the variance, respectively (Figure 7B). Paired samples demonstrated shifts in metabolomic profiles between active NEC and subsequent reanastomosis, although the small number of paired specimens precludes definitive conclusions regarding longitudinal changes.

### 3.9. NEC-Associated Metabolite Differences Persist Across Surgical Control Groups

**Figure 8:**
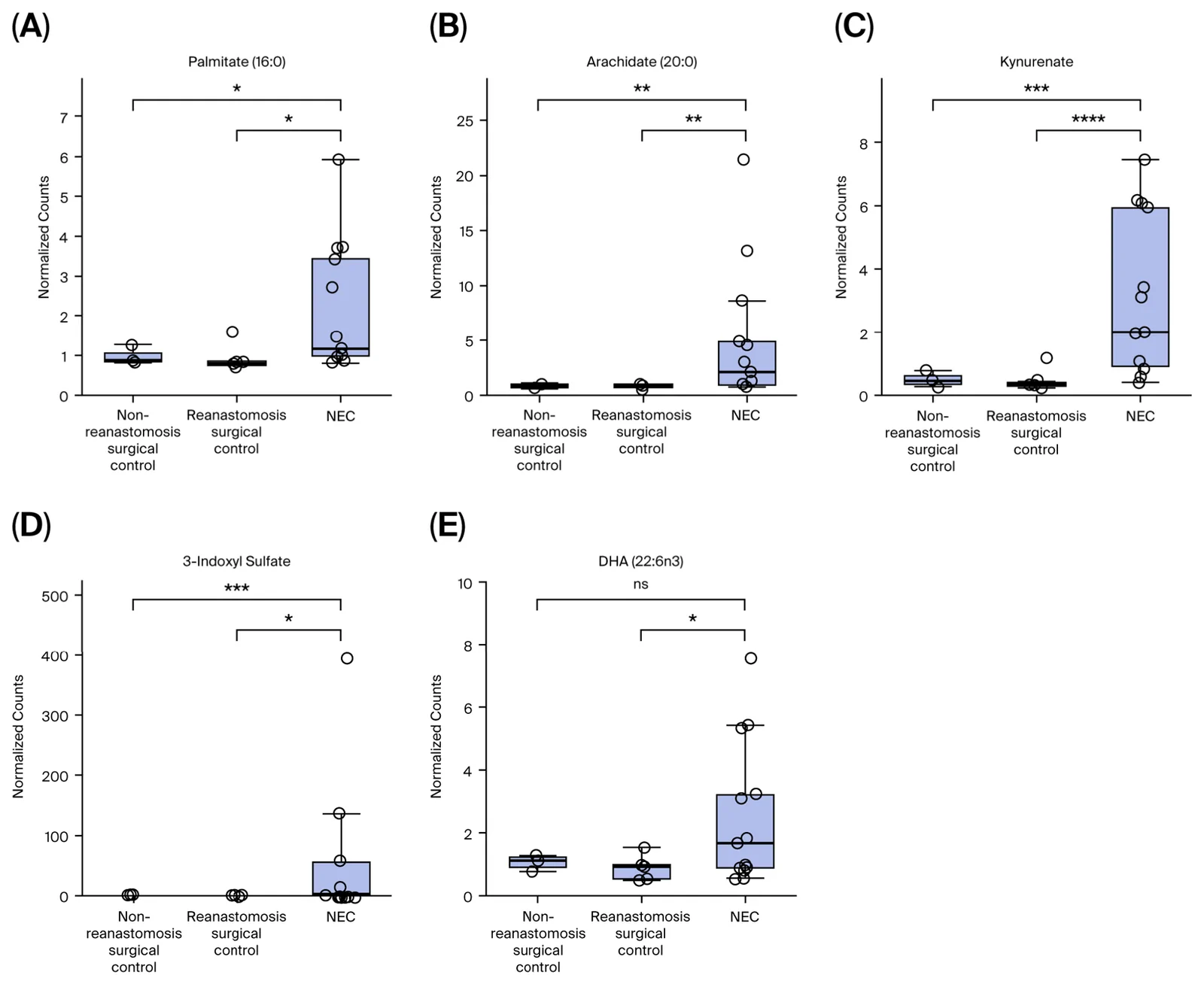
Normalized boxplots of representative metabolites comparing intestinal tissue from infants with NEC with surgical control samples stratified by surgical context. Metabolites displayed include (**A**) palmitate, (**B**) arachidate, (**C**) kynurenate, (**D**) 3-indoxyl sulfate, and (**E**) DHA. Surgical controls included samples collected at the time of reanastomosis (n = 5) and samples collected during other surgical procedures without reanastomosis (n = 3). * = *p* < 0.05; ** = *p* < 0.01; *** = *p* < 0.001; **** = *p* < 0.0001; ns = *p* ≥ 0.05.

Normalized boxplots were constructed for key metabolites to visualize whether NEC-associated differences persisted when the surgical control groups were evaluated separately (Figure 8). Representative metabolites that were significant in the primary analysis, including palmitate, arachidate, kynurenate, and 3-indoxyl sulfate, remained significantly different between NEC and both surgical control groups. DHA remained significantly different between NEC and reanastomosis controls but did not reach statistical significance when NEC was compared with non-reanastomosis surgical controls. Together, these findings indicate that the major NEC-associated metabolite differences were generally preserved when the two surgical control groups were evaluated separately.

### 3.10. NEC-Associated Metabolite Differences Persist in Small-Intestinal-Only Analysis

**Figure 9:**
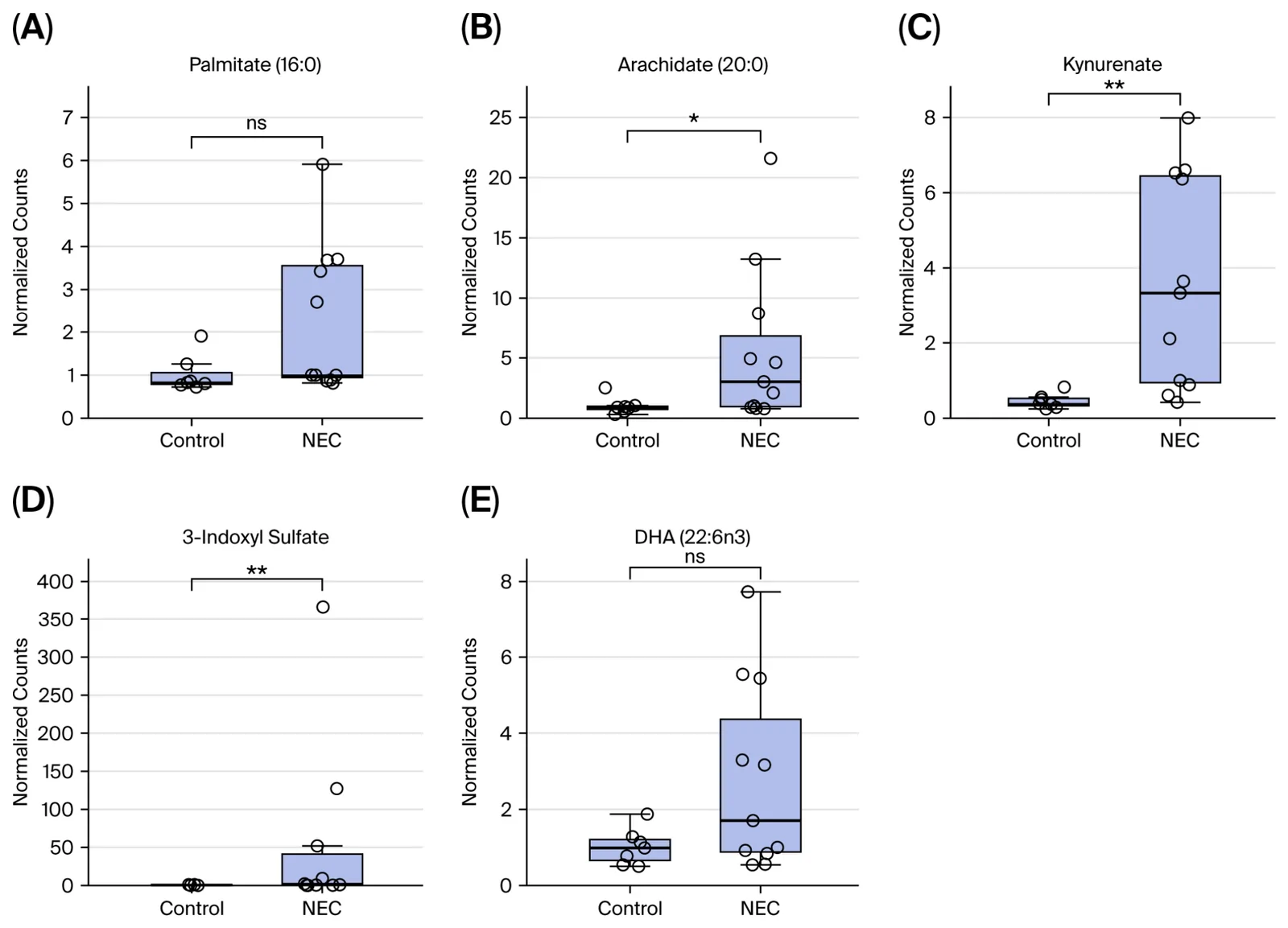
NEC-associated metabolite differences in small-intestinal tissue. Metabolites displayed include (**A**) palmitate, (**B**) arachidate, (**C**) kynurenate, (**D**) 3-indoxyl sulfate, and (**E**) DHA. Normalized box plots of representative metabolites following exclusion of colonic samples. The small-intestine-only analysis included 11 NEC and 7 control samples. Statistical comparisons were performed using mixed-effects models and individual points represent tissue samples. * = *p* < 0.05; ** = *p* < 0.01; ns = *p* ≥ 0.05.

To evaluate whether differences in intestinal anatomic location contributed to the observed metabolomic differences, we performed a sensitivity analysis excluding all colonic samples. The small-intestine-only cohort included 11 NEC and 7 control samples. Using the same mixed-effects modeling approach as the primary analysis, arachidate (*p* = 0.014), kynurenate (*p* = 0.0077), and 3-indoxyl sulfate (*p* = 0.0081) remained significantly different between NEC and control tissue (Figure 9). Palmitate (*p* = 0.071) and DHA (*p* = 0.080) did not reach statistical significance after exclusion of colonic samples. These findings demonstrate that several of the major NEC-associated metabolite differences persisted when the analysis was restricted to small-intestinal tissue.

Given the limited number of colonic samples, regional differences were examined descriptively. The two NEC colonic samples generally fell within the range of metabolite abundances observed in NEC small-intestinal samples for the representative metabolites examined. Similarly, the single colonic control fell within the small-intestinal control range for most metabolites, although kynurenate was higher and 3-indoxyl sulfate was not detected.

## 4. Discussion

To the best of our knowledge, this is the first study examining the metabolome of intestinal tissue in NEC patients compared with gestational and age-matched preterm infants undergoing surgery for indications other than NEC. Previous urinary, fecal, and blood metabolome studies have not identified a metabolite signature or biomarker that consistently predicts or diagnoses NEC, and the underlying metabolic mechanisms remain unclear [11,19]. Using tissue samples may more accurately represent the site of local injury during NEC. By establishing interinstitutional collaborations and regular communication with pediatric surgery, we collected fresh intestinal tissue at the time of surgery for both NEC and surgical controls. We discovered several significant differences in compound production or depletion between control and NEC patients, reflective of tissue-level damage, and likely secondary to alterations in the gut microbiome known to occur with NEC and associated changes in microbe and host-metabolite production [8,20,21].

One of the more clinically meaningful findings in this study is the marked increase in indole-containing compounds, including an approximately 11-fold elevation of indoxyl sulfate (IS) in NEC patients, which was nominally significant. IS is a host–microbial co-metabolite generated following bacterial metabolism of tryptophan to indole, which is subsequently absorbed and metabolized by the host to form IS [22]. Importantly, IS is primarily excreted by the kidneys, and elevated tissue concentrations may therefore reflect alterations in microbial precursor production, host metabolism, renal clearance, or a combination of these processes. IS has been implicated in intestinal barrier dysfunction in human cell lines and mouse models [23]. Studies examining the effect of IS on intestinal epithelial cells (IECs) reported a significant increase in the production of reactive oxygen species (ROS) in IECs with elevated serum IS levels [24]. IS may also have a negative effect on intestinal barrier integrity likely due to its suppression of tight junction-related genes in IECs. Lastly, IS is known to inhibit mitophagy- the process by which damaged mitochondria are selectively degraded- by downregulating dynamin-related protein 1 (DRP-1) expression. This inhibition leads to the accumulation of damaged mitochondria, heightened ROS production, and subsequent cellular injury [25]. Thus, intestinal injury generated by IS, including ROS, leaky intestinal barrier, and impaired clearance of old mitochondria, overlap with mechanisms responsible for NEC and raise the possibility that elevated amounts of IS may contribute to intestinal injury. However, IS was only nominally significant and did not remain significant after corrections for multiple testing; therefore, these findings should be interpreted cautiously. Further studies incorporating microbiome and renal function data will be necessary to determine the source and potential biological significance of elevated IS in NEC.

Our findings suggest a dual role for fatty acid metabolites in modulating intestinal inflammation and barrier integrity, with some metabolites promoting inflammation and injury while others exert protective and anti-inflammatory effects. Among these, the long-chain saturated fatty acid palmitate was approximately 2-fold higher in NEC tissue compared with control tissue. On an intestinal level, palmitate, which has been demonstrated to play a role in chronic liver disease, diabetes, and Crohn’s disease, is known to activate monocytes and stimulates proinflammatory responses in human immune cells [26,27,28]. In intestinal epithelial cells, palmitate has been shown to increase intestinal permeability through alterations in tight junction and adherens junction protein complexes, suggesting a potential mechanism by which elevated palmitate could contribute to the impaired intestinal barrier characteristic of NEC [29,30]. However, palmitate did not remain significant after FDR correction, limiting our ability to draw mechanistic conclusions from this finding. The relative abundance of arachidate was also three-fold higher in NEC tissue. This long-chain saturated free fatty acid has been implicated in intestinal barrier dysfunction, including increased intestinal permeability and alterations in villus morphology. Arachidate has also been associated with gut microbial dysbiosis, including enrichment of *Lachnospiraceae*, suggesting potential effects on both epithelial barrier integrity and the intestinal microbial environment [31,32].

Conversely, while docosahexaenoate (DHA) abundance did not differ significantly between NEC and controls in the primary analysis, DHA was significantly increased in NEC tissue compared with surgical control samples collected following reanastomosis. This finding may reflect altered DHA metabolism or mobilization from membrane phospholipids in the setting of intestinal inflammation. DHA has been shown to attenuate oxidative stress by reducing reactive oxygen species production, enhancing antioxidant enzyme activity, and improving overall antioxidant capacity in inflammatory conditions [33,34]. Given these anti-inflammatory and antioxidant properties, enteral DHA supplementation has been investigated as a potential strategy to reduce the risk of NEC in preterm infants [35]. In addition, animal studies suggest that maternal DHA supplementation during gestation may attenuate LPS-induced fetal growth restriction [36]. Collectively, our observations suggest that altered tissue DHA abundance may accompany the inflammatory and oxidative changes associated with NEC, although the present study cannot determine whether this represents a protective response or a consequence of tissue injury.

Marked elevation of steroid metabolites in NEC tissue, specifically progestin steroids, may reflect multifactorial disturbances in steroid metabolism and signaling. Preterm infants, particularly those with NEC, have immature hepatic enzyme systems and may exhibit impaired metabolic capacity within the intestinal mucosa, both of which are critical for steroid clearance. Inflammation-associated cholestasis, as may be seen in NEC, may further compromise hepatic metabolism, contributing to the accumulation of steroid metabolites. Elevated progestin levels in NEC tissue could also represent a systemic or local stress response. Alternatively, increased synthesis of progestin steroids—known to enhance epithelial barrier integrity and suppress pro-inflammatory cytokine production—may represent a compensatory mechanism to mitigate intestinal injury [37]. Finally, NEC patients may have been more likely to be exposed to stress-dose hydrocortisone; however, data on steroid use in NEC versus control patients were not collected in this study.

Retinol metabolism emerged as the most significantly enriched metabolic pathway in NEC intestinal tissue, suggesting disruption of vitamin A–dependent pathways involved in epithelial differentiation, mucosal barrier maintenance, and immune regulation. Given the established role of retinoid signaling in intestinal homeostasis and repair, these findings raise the possibility that altered retinol metabolism contributes to impaired epithelial regeneration during NEC [38,39,40].

The striking increase in caffeine and other xanthine metabolites in NEC samples is notable, as these compounds are known to decrease mesenteric blood flow in a dose-dependent manner, potentiating intestinal ischemia [41,42,43]. Caffeine therapy is a cornerstone in the management of apnea of prematurity and ranks among the five most frequently prescribed medications in neonatology [44,45]. Because there was no statistically significant difference in gestational or chronological ages between the two groups, exposure to caffeine therapy is expected to have been comparable. However, exogenous administration cannot be excluded as a contributor to the elevated metabolite levels detected. Further studies examining the dosing, timing, and pharmacokinetics of caffeine may help clarify potential relationships between mesenteric perfusion and subsequent intestinal injury.

The strength of our study was the unique access to human tissue samples, which may be the best representation of the metabolomic milieu at the site of injury. Another strength was the existence of a comparison group that was well matched for important modifiers like chronologic and gestational age, mode of delivery, feeding type, and antibiotic exposure. However, comprehensive clinical data were not available for all patients, limiting our ability to evaluate the potential influence of additional clinical variables, including medication exposures, on the observed metabolomic differences. Other limitations are the small size and biological heterogeneity of the surgical control group, which included intestinal tissue obtained from infants with SIP, volvulus, and during ostomy takedown/reanastomosis. These samples therefore represent clinically relevant surgical comparators rather than normal healthy intestinal tissue. To address this limitation, we performed secondary analyses stratifying the control samples by surgical context, comparing active NEC with non-ostomy surgical controls and ostomy takedown/reanastomosis controls. In these secondary analyses, key NEC-associated metabolites generally remained significantly different when the two surgical control groups were evaluated separately, suggesting that the observed NEC-associated metabolomic differences were not solely attributable to heterogeneity within the control group.

Tissue composition was also not independently quantified, and variability in the cellular and structural composition of the surgical specimens may have influenced the observed metabolomic profiles. Another limitation is the absence of concomitant microbiome data alongside the tissue metabolome. This was primarily due to the quantity of starting material and the tissue requirements for metabolomic studies. However, some major bacterial classes can be inferred based on our understanding of bacterial metabolite production and substrate interactions in the intestines. For example, indole-containing compounds, which were more frequently detected in NEC samples, are predominantly produced by intestinal bacteria from the Proteobacteria phylum, including *Escherichia coli* and *Proteus* species [46]. This finding may reflect alterations in microbial tryptophan metabolism associated with NEC and is consistent with a Proteobacteria-dominant dysbiosis previously described in NEC [47,48]. Similarly, 4-hydroxyphenylacetate, a product of microbial tyrosine metabolism produced by several intestinal bacteria, including *Bacteroides* species, was increased 3.2-fold in NEC tissue. This finding further supports alterations in microbial aromatic amino acid metabolism in NEC. Future studies integrating metabolite profiling with paired microbiome sequencing will clarify the potential relevance of these metabolic changes to NEC pathophysiology.

Taken together, these findings demonstrate the distinct tissue-level metabolites in control and NEC patients, many of which have the potential to adversely affect intestinal integrity. Several of the derangements in metabolites in NEC patients have not been detected previously in urine, fecal, or blood metabolomic studies and may lead to novel hypotheses on the pathogenesis of NEC that can be tested in future studies.

## Figures and Tables

**Table 1:** Cohort Characteristics.

	Control	NEC	*p*-Value ^2^
	n = 8	n = 13	
Gestational age (weeks), mean ± SD	28± 5	28± 2	1.0
Birthweight (grams), mean ± SD	1177 ± 726	947 ± 467	0.44
Post menstrual age at surgery, mean ± SD	35± 6	29± 9	0.13
Male, % (n)	62 (5)	61 (8)	0.97
Day of life of surgery, mean ± SD	51± 41	24± 18	0.11
Mode of delivery (Cesarean section), % (n)	62 (5)	61 (8)	0.96
Breastmilk enteral feeds, % (n)	38 (3)	54 (7)	0.72
Early antibiotics (within first 48 h, % (n)	100 (8)	100 (13)	n/a ^1^
Perioperative antibiotics (>24 h prior to surgery, % (n)	62 (5)	54 (7)	1.0

^1^ *p* value not applicable due to no variation in antibiotic use across groups. ^2^ Statistical analyses were conducted using Fisher exact or chi-squared tests for categorical variables and Welch’s *t*-test for continuous variables.

## Data Availability

The original contributions presented in this study are included in the article and Supplementary Materials. Metabolomics data is available at the NIH Common Fund’s National Metabolomics Data Repository (NMDR) website, the Metabolomics Workbench, https://www.metabolomicsworkbench.org, accessed on 21 September 2026, where it has been assigned Project ID: ST005211. The data can be accessed directly via its Project DOI: http://dx.doi.org/10.21228/M8W00Z. This work is supported by Metabolomics Workbench/National Metabolomics Data Repository (NMDR) (grant# U2C-DK119886), Common Fund Data Ecosystem (CFDE) (grant# 3OT2OD030544) and Metabolomics Consortium Coordinating Center (M3C) (grant# 1U2C-DK119889).

## Supplementary Materials

The following supporting information can be downloaded at https://www.mdpi.com/article/10.3390/microorganisms14102202/s1, Table S1: Differentially abundant metabolites in intestinal tissues from infants with necrotizing enterocolitis (NEC) and non-NEC controls. Table S2: Detection frequencies of annotated metabolites with nominally significant differential abundance in NEC intestinal tissue.
