# Supplementary material for: Untargeted Metabolomic Profiling Reveals a Distinct Intestinal Metabolic Signature in Necrotizing Enterocolitis
Source: Microorganisms. 2026 Oct 1;14(10):2202. doi: 10.3390/microorganisms14102202 (PMC13707595; doi:10.3390/microorganisms14102202)
Supplement: Supplementary file 1 [file microorganisms-14-02202-s001.zip › microorganisms-4505834-supplementary.pdf]

Supplementary Table S1. Differentially abundant metabolites in intestinal tissues from infants with necrotizing enterocolitis (NEC) and non-NEC controls.

Untargeted metabolomic profiling identified 1,183 biochemical features. Using a linear mixed-effects model with disease status as a fixed effect and patient as a random effect to account for repeated samples, 308 metabolites were nominally significant at $P < 0.05$. The 255 annotated metabolites are shown below; unidentified metabolites are excluded. Fold change is NEC/control; values >1 indicate higher abundance in NEC and values <1 indicate lower abundance in NEC. Ninety-five percent confidence intervals were calculated on the log2 abundance scale and back-transformed to the fold-change scale. Q values are Benjamini-Hochberg FDR-adjusted across all metabolites with estimable P values (n=998). Missing measurements were omitted metabolite-by-metabolite.

Metabolite	Fold Change (NEC/Control)	95% CI	P value	Q value
**AMINO ACID**				
**Glycine, Serine and Threonine Metabolism**				
betaine aldehyde	0.150	0.058–0.392	<0.001	0.001
**Histidine Metabolism**				
1-methylhistidine	2.002	1.467–2.732	<0.001	<0.001
N-acetylhistidine	3.376	2.153–5.294	<0.001	<0.001
imidazole propionate	3.789	1.628–8.817	0.002	0.016
imidazole lactate	6.379	2.548–15.969	<0.001	<0.001
histamine	0.079	0.026–0.243	<0.001	<0.001
1-methylhistamine	0.408	0.179–0.929	0.033	0.138
1-ribosyl-imidazoleacetate*	2.259	1.128–4.521	0.021	0.101
N-acetylhistamine	0.362	0.297–0.440	<0.001	<0.001
**Lysine Metabolism**				
N2-acetyl,N6,N6-dimethyllysine	2.528	1.336–4.781	0.004	0.030
pipecolate	2.455	1.220–4.944	0.015	0.077
cadaverine	33.043	6.605–165.313	<0.001	<0.001
**Phenylalanine Metabolism**				
N-acetylphenylalanine	2.235	1.242–4.023	0.007	0.044
phenyllactate (PLA)	5.208	3.380–8.025	<0.001	<0.001
4-hydroxyphenylacetate	3.219	2.766–3.745	<0.001	<0.001
**Tyrosine Metabolism**				
3-(4-hydroxyphenyl)lactate	3.451	1.200–9.919	0.021	0.101
4-methoxyphenol sulfate	7.044	2.819–17.601	<0.001	<0.001
vanillactate	6.606	4.713–9.261	<0.001	<0.001
vanillylmandelate (VMA)	2.082	1.002–4.326	0.050	0.182
3-methoxytyramine sulfate	3.646	1.211–10.981	0.021	0.101
homovanillate sulfate	8.290	2.449–28.066	0.002	0.016
tyramine O-sulfate	1.838	1.603–2.108	<0.001	<0.001
vanillic alcohol sulfate	1.474	1.359–1.598	<0.001	<0.001
**Tryptophan Metabolism**				
oxindolylalanine	2.129	1.202–3.770	0.010	0.055
tryptophan betaine	2.329	1.627–3.333	<0.001	<0.001
N-acetylkynurenine (2)	5.209	1.685–16.100	0.004	0.029
kynurenate	4.705	2.845–7.781	<0.001	<0.001
8-methoxykynurenate	3.556	1.764–7.167	<0.001	0.004
5-hydroxyindoleacetate	2.512	2.328–2.710	<0.001	<0.001
indolelactate	2.665	1.746–4.066	<0.001	<0.001
indole-3-carboxylate	2.575	1.893–3.501	<0.001	<0.001
3-indoxyl sulfate	11.172	1.334–93.584	0.026	0.117
**Leucine, Isoleucine and Valine Metabolism**				
N-acetylleucine	2.356	1.384–4.011	0.002	0.013
alpha-hydroxyisocaproate	3.788	1.800–7.969	<0.001	0.004
isovalerylglycine	1.100	1.034–1.170	0.003	0.020

Metabolite	Fold Change (NEC/Control)	95% CI	P value	Q value
beta-hydroxyisovalerate	2.626	1.387–4.973	0.003	0.022
3-methylglutaconate	2.382	1.170–4.850	0.017	0.083
N-acetylisoleucine	2.284	1.418–3.680	<0.001	0.006
2-hydroxy-3-methylvalerate	5.617	1.963–16.071	0.001	0.011
N-acetylvaline	1.567	1.120–2.194	0.009	0.052
alpha-hydroxyisovalerate	5.355	2.677–10.712	<0.001	<0.001
3-hydroxyisobutyrate	2.332	1.817–2.993	<0.001	<0.001
**Methionine, Cysteine, SAM and Taurine Metabolism**				
2,3-dihydroxy-5-methylthio-4-pentenoate (DMTPA)*	2.564	1.550–4.242	<0.001	0.002
S-methylcysteine sulfoxide	0.318	0.161–0.625	<0.001	0.008
cysteine sulfinic acid	0.427	0.234–0.778	0.005	0.036
N-acetyltaurine	1.910	1.310–2.785	<0.001	0.007
**Urea cycle; Arginine and Proline Metabolism**				
urea	3.808	2.774–5.227	<0.001	<0.001
N-acetylproline	0.464	0.464–0.464	<0.001	<0.001
N-methylproline	2.344	1.001–5.486	0.050	0.182
N-monomethylarginine	0.524	0.275–0.998	0.049	0.182
**Creatine Metabolism**				
guanidinoacetate	0.339	0.158–0.728	0.006	0.036
creatinine	1.709	1.143–2.555	0.009	0.052
**Polyamine Metabolism**				
N-acetylputrescine	2.744	1.925–3.912	<0.001	<0.001
N-acetyl-isoputreanine	2.705	1.479–4.944	0.001	0.010
diacetylspermidine*	3.986	1.683–9.440	0.004	0.029
(N(1) + N(8))-acetylspermidine	4.049	1.928–8.503	<0.001	0.002
spermine	0.519	0.276–0.978	0.042	0.166
N(1)-acetylspermine	4.984	1.175–21.142	0.029	0.127
N1,N12-diacetylspermine	14.280	3.733–54.634	<0.001	0.001
5-methylthioadenosine (MTA)	3.733	1.275–10.929	0.016	0.082
4-acetamidobutanoate	4.521	4.244–4.815	<0.001	<0.001
**Glutathione Metabolism**				
glutathione, oxidized (GSSG)	1.689	1.394–2.046	<0.001	<0.001
2-hydroxybutyrate/2-hydroxyisobutyrate	2.747	1.363–5.537	0.005	0.031
**PEPTIDE**				
**Gamma-glutamyl Amino Acid**				
gamma-glutamylalanine	1.498	1.307–1.716	<0.001	<0.001
gamma-glutamylhistidine	1.803	1.498–2.170	<0.001	<0.001
gamma-glutamylvaline	1.952	1.781–2.139	<0.001	<0.001
**Dipeptide**				
cyclo(leu-pro)	3.379	3.378–3.381	<0.001	<0.001
glycylleucine	3.039	1.044–8.847	0.041	0.165
leucylalanine	2.515	1.819–3.477	<0.001	<0.001
lysylleucine	1.627	1.301–2.033	<0.001	<0.001
threonylphenylalanine	6.570	3.346–12.903	<0.001	<0.001
valylglycine	2.100	1.152–3.828	0.015	0.079
valylleucine	2.383	1.032–5.501	0.042	0.165
leucylglutamine*	2.264	1.024–5.007	0.044	0.169
**Acetylated Peptides**				
phenylacetylglutamine	5.774	2.243–14.867	<0.001	0.003
**Modified Peptides**				
pyroglutamylglycine	0.535	0.309–0.925	0.025	0.113
**CARBOHYDRATE**				

Metabolite	Fold Change (NEC/Control)	95% CI	P value	Q value
**Glycolysis, Gluconeogenesis, and Pyruvate Metabolism**				
fructose 6-phosphate	2.989	1.051–8.500	0.040	0.160
2,3-diphosphoglycerate	0.516	0.299–0.890	0.017	0.086
**Pentose Phosphate Pathway**				
ribose 1-phosphate	1.842	1.277–2.655	0.001	0.009
sedoheptulose-7-phosphate	2.640	1.322–5.271	0.006	0.038
**Glycogen Metabolism**				
maltose	0.603	0.382–0.952	0.030	0.128
**Fructose, Mannose and Galactose Metabolism**				
galactose 1-phosphate	1.290	1.205–1.382	<0.001	<0.001
**Nucleotide Sugar**				
UDP-glucose	0.076	0.008–0.720	0.025	0.112
UDP-galactose	0.131	0.045–0.388	<0.001	0.002
UDP-N-acetylglucosamine/galactosamine	3.260	1.003–10.597	0.049	0.182
**Aminosugar Metabolism**				
erythronate*	2.002	1.244–3.224	0.004	0.029
**LIPID**				
**Medium Chain Fatty Acid**				
heptanoate (7:0)	0.531	0.291–0.969	0.039	0.158
**Long Chain Saturated Fatty Acid**				
palmitate (16:0)	1.811	1.048–3.129	0.035	0.144
margarate (17:0)	3.518	1.358–9.109	0.010	0.055
stearate (18:0)	2.029	1.267–3.248	0.003	0.023
nonadecanoate (19:0)	3.601	1.311–9.888	0.013	0.069
arachidate (20:0)	3.001	1.357–6.638	0.007	0.041
**Long Chain Monounsaturated Fatty Acid**				
palmitoleate (16:1n7)	3.050	1.106–8.411	0.031	0.133
heptadecenoate (17:1)	3.574	1.275–10.017	0.015	0.079
oleate/vaccenate (18:1)	2.568	1.147–5.750	0.024	0.111
nonadecenoate (19:1)	3.499	1.638–7.471	0.001	0.010
eicosenoate (20:1)	5.355	1.687–16.998	0.004	0.030
erucate (22:1n9)	4.713	1.772–12.534	0.002	0.015
**Long Chain Polyunsaturated Fatty Acid (n3 and n6)**				
docosatrienoate (22:3n3)	4.077	1.108–15.004	0.035	0.143
linoleate (18:2n6)	2.897	1.081–7.763	0.034	0.143
dihomo-linoleate (20:2n6)	4.556	1.295–16.028	0.018	0.089
arachidonate (20:4n6)	2.309	1.169–4.563	0.016	0.082
adrenate (22:4n6)	4.829	1.582–14.741	0.006	0.037
docosapentaenoate (n6 DPA; 22:5n6)	3.935	1.440–10.747	0.008	0.046
docosadienoate (22:2n6)	4.969	1.387–17.804	0.014	0.073
**Fatty Acid, Branched**				
(16 or 17)-methylstearate (a19:0 or i19:0)	3.239	1.118–9.385	0.030	0.130
**Fatty Acid, Dicarboxylate**				
2-hydroxyadipate	1.423	1.423–1.423	<0.001	<0.001
tetradecanedioate (C14-DC)	0.364	0.221–0.599	<0.001	<0.001
hexadecanedioate (C16-DC)	0.542	0.304–0.966	0.038	0.155
hexadecenedioate (C16:1-DC)*	0.449	0.213–0.948	0.036	0.146
**Fatty Acid, Amino**				
N-acetyl-2-aminooctanoate*	1.524	1.140–2.037	0.004	0.030
**Fatty Acid Metabolism (Acyl Glycine)**				
valerylglycine	1.269	1.147–1.404	<0.001	<0.001
**Fatty Acid Metabolism (Acyl Carnitine, Long Chain Saturated)**				
myristoylcarnitine (C14)	0.610	0.413–0.900	0.013	0.068

Metabolite	Fold Change (NEC/Control)	95% CI	P value	Q value
**Fatty Acid Metabolism (Acyl Carnitine, Monounsaturated)**				
palmitoleoylcarnitine (C16:1)*	0.514	0.290–0.911	0.023	0.105
**Fatty Acid Metabolism (Acyl Carnitine, Dicarboxylate)**				
octadecenedioylcarnitine (C18:1-DC)*	0.796	0.750–0.846	<0.001	<0.001
**Fatty Acid Metabolism (Acyl Carnitine, Hydroxy)**				
3-hydroxypalmitoylcarnitine	0.515	0.298–0.891	0.018	0.087
**Fatty Acid, Monohydroxy**				
2-hydroxyarachidate*	2.689	1.320–5.477	0.006	0.040
2-hydroxynervonate*	2.952	1.320–6.601	0.008	0.049
3-hydroxyoctanoate	0.729	0.572–0.929	0.011	0.060
3-hydroxydecanoate	0.676	0.500–0.913	0.011	0.061
**Fatty Acid, Dihydroxy**				
12,13-DiHOME	3.917	1.436–10.684	0.008	0.046
2,4-dihydroxybutyrate	1.856	1.031–3.342	0.039	0.158
**Eicosanoid**				
5-HETE	3.392	2.214–5.196	<0.001	<0.001
**Endocannabinoid**				
N-oleoyltaurine	3.339	1.080–10.325	0.036	0.149
N-stearoyltaurine	1.992	1.056–3.759	0.033	0.140
linolenoyl ethanolamide (18:3)*	6.038	5.292–6.868	<0.001	<0.001
N-oleoylserine	2.279	1.137–4.568	0.020	0.097
**Phospholipid Metabolism**				
glycerophosphoethanolamine	1.837	1.108–3.047	0.019	0.090
**Phosphatidylcholine (PC)**				
1-palmitoyl-2-pentadecanoyl-GPC (16:0/15:0)*	0.550	0.306–0.992	0.047	0.178
1-palmitoyl-2-linoleoyl-GPC (16:0/18:2)	0.598	0.359–0.997	0.049	0.182
1-palmitoyl-2-eicosapentaenoyl-GPC (16:0/20:5)*	0.324	0.106–0.995	0.049	0.182
1-palmitoyl-2-docosahexaenoyl-GPC (16:0/22:6)	0.435	0.431–0.439	<0.001	<0.001
1-stearoyl-2-linoleoyl-GPC (18:0/18:2)*	0.465	0.222–0.973	0.042	0.166
1,2-dioleoyl-GPC (18:1/18:1)	0.913	0.895–0.931	<0.001	<0.001
1,2-dilinoleoyl-GPC (18:2/18:2)	0.351	0.179–0.690	0.002	0.018
**Phosphatidylethanolamine (PE)**				
1-palmitoyl-2-linoleoyl-GPE (16:0/18:2)	0.379	0.219–0.655	<0.001	0.005
1-palmitoyl-2-eicosapentaenoyl-GPE (16:0/20:5)*	0.302	0.106–0.860	0.025	0.113
1-stearoyl-2-oleoyl-GPE (18:0/18:1)	0.669	0.474–0.945	0.023	0.105
1-stearoyl-2-dihomo-linolenoyl-GPE (18:0/20:3n3 or n6)*	0.400	0.227–0.704	0.001	0.012
1-stearoyl-2-docosahexaenoyl-GPE (18:0/22:6)*	0.276	0.274–0.277	<0.001	<0.001
**Phosphatidylserine (PS)**				
1-stearoyl-2-oleoyl-GPS (18:0/18:1)	0.516	0.290–0.917	0.024	0.111
1-stearoyl-2-docosahexaenoyl-GPS (18:0/22:6)*	0.375	0.239–0.589	<0.001	<0.001
**Lysophospholipid**				
1-palmitoleoyl-GPC (16:1)*	0.700	0.510–0.961	0.027	0.120
1-palmitoyl-GPE (16:0)	1.227	1.022–1.473	0.028	0.124
2-stearoyl-GPE (18:0)*	1.444	1.003–2.078	0.048	0.180
2-arachidonoyl-GPE (20:4)*	1.798	1.005–3.215	0.048	0.180
1-palmitoyl-GPS (16:0)*	3.556	1.265–10.000	0.016	0.082
1-oleoyl-GPS (18:1)	2.377	1.020–5.540	0.045	0.173
1-oleoyl-GPG (18:1)*	2.329	1.245–4.356	0.008	0.049
1-oleoyl-GPI (18:1)	2.590	1.347–4.978	0.004	0.030
1-arachidonoyl-GPI (20:4)*	1.494	1.082–2.063	0.015	0.077
**Plasmalogen**				
1-(1-enyl-palmitoyl)-2-oleoyl-GPE (P-16:0/18:1)*	0.536	0.352–0.817	0.004	0.027

Metabolite	Fold Change (NEC/Control)	95% CI	P value	Q value
1-(1-enyl-palmitoyl)-2-linoleoyl-GPE (P-16:0/18:2)*	0.268	0.137–0.524	<0.001	0.001
1-(1-enyl-stearoyl)-2-oleoyl-GPE (P-18:0/18:1)	0.753	0.570–0.995	0.046	0.175
1-(1-enyl-palmitoyl)-2-arachidonoyl-GPC (P-16:0/20:4)*	1.377	1.011–1.877	0.042	0.166
**Lysoplasmalogen**				
1-palmityl-GPE (O-16:0)*	5.017	1.938–12.993	<0.001	0.008
1-stearyl-GPE (O-18:0)*	3.165	1.663–6.025	<0.001	0.004
1-(1-enyl-stearoyl)-GPE (P-18:0)*	2.481	1.066–5.779	0.035	0.145
**Monoacylglycerol**				
1-myristoylglycerol (14:0)	7.561	1.554–36.789	0.012	0.067
1-palmitoylglycerol (16:0)	6.076	1.577–23.421	0.009	0.052
1-palmitoleoylglycerol (16:1)*	6.369	1.356–29.918	0.019	0.092
1-linoleoylglycerol (18:2)	5.913	1.069–32.703	0.042	0.165
1-arachidonylglycerol (20:4)	4.910	1.128–21.363	0.034	0.142
1-docosahexaenoylglycerol (22:6)	5.058	1.156–22.118	0.031	0.133
2-palmitoylglycerol (16:0)	2.093	1.852–2.367	<0.001	<0.001
2-palmitoleoylglycerol (16:1)*	3.535	1.147–10.898	0.028	0.122
2-oleoylglycerol (18:1)	6.595	2.409–18.056	<0.001	0.002
2-linoleoylglycerol (18:2)	5.153	1.270–20.912	0.022	0.102
**Diacylglycerol**				
palmitoyl-linolenoyl-glycerol (16:0/18:3) [2]*	3.321	1.481–7.446	0.004	0.026
palmitoyl-arachidonoyl-glycerol (16:0/20:4) [2]*	2.252	1.193–4.252	0.012	0.067
oleoyl-linoleoyl-glycerol (18:1/18:2) [1]	3.044	1.391–6.660	0.005	0.035
linoleoyl-linoleoyl-glycerol (18:2/18:2) [1]*	5.997	1.858–19.363	0.003	0.020
oleoyl-arachidonoyl-glycerol (18:1/20:4) [1]*	0.555	0.513–0.600	<0.001	<0.001
**Galactosyl Glycerolipids**				
galactosylglycerol	3.580	1.262–10.155	0.016	0.083
**Sphingolipid Synthesis**				
sphingadienine	1.867	1.362–2.560	<0.001	0.001
**Dihydroceramides**				
N-stearoyl-sphinganine (d18:0/18:0)*	1.946	1.666–2.274	<0.001	<0.001
**Hexosylceramides (HCER)**				
glycosyl-N-stearoyl-sphingosine (d18:1/18:0)	0.539	0.538–0.539	<0.001	<0.001
**Dihydrosphingomyelins**				
sphingomyelin (d18:0/20:0, d16:0/22:0)*	1.531	1.075–2.181	0.018	0.089
**Mevalonate Metabolism**				
3-hydroxy-3-methylglutarate	1.611	1.023–2.537	0.040	0.159
mevalonate	1.974	1.020–3.821	0.044	0.169
**Sterol**				
4-cholesten-3-one	1.826	1.706–1.954	<0.001	<0.001
7-hydroxycholesterol (alpha or beta)	3.117	1.044–9.303	0.042	0.165
**Pregnenolone Steroids**				
pregnenolone sulfate	7.691	3.138–18.853	<0.001	<0.001
17alpha-hydroxypregnenolone 3-sulfate	5.199	2.276–11.878	<0.001	0.001
21-hydroxypregnenolone disulfate	8.999	3.110–26.038	<0.001	<0.001
pregnenediol sulfate (C21H34O5S)*	12.488	4.300–36.266	<0.001	<0.001
pregnenediol disulfate (C21H34O8S2)*	16.633	5.130–53.929	<0.001	<0.001
pregnenetriol sulfate*	3.537	1.322–9.466	0.012	0.065
pregnenetriol disulfate*	9.870	3.969–24.545	<0.001	<0.001
**Progestin Steroids**				
5alpha-pregnan-3beta,20alpha-diol disulfate	22.481	2.027–249.270	0.011	0.063
**Corticosteroids**				
cortisol	3.957	1.042–15.033	0.044	0.170

Metabolite	Fold Change (NEC/Control)	95% CI	P value	Q value
cortisol 21-sulfate	6.845	1.637–28.633	0.013	0.068
cortisone	1.854	1.276–2.692	0.001	0.010
**Androgenic Steroids**				
dehydroepiandrosterone sulfate (DHEA-S)	7.772	3.251–18.581	<0.001	<0.001
16alpha-hydroxy DHEA 3-sulfate	3.465	1.354–8.867	0.010	0.055
androstenediol (3beta,17beta) disulfate (1)	13.152	2.408–71.826	0.003	0.022
androstenediol (3beta,17beta) disulfate (2)	7.722	1.592–37.445	0.011	0.063
andro steroid monosulfate C19H28O6S (1)*	10.075	2.427–41.826	0.001	0.012
**Primary Bile Acid Metabolism**				
tauro-alpha-muricholate	0.232	0.055–0.981	0.047	0.178
**Secondary Bile Acid Metabolism**				
glycoursodeoxycholate	0.051	0.007–0.403	0.005	0.031
taurohyodeoxycholic acid	1.791	1.270–2.526	<0.001	0.008
glycocholenate sulfate*	0.292	0.086–0.996	0.049	0.182
**NUCLEOTIDE**				
**Purine Metabolism, Adenine containing**				
N6-carbamoylthreonyladenosine	1.647	1.093–2.480	0.017	0.085
2'-deoxyadenosine	0.825	0.791–0.862	<0.001	<0.001
**Purine Metabolism, Guanine containing**				
7-methylguanine	2.480	1.663–3.697	<0.001	<0.001
N2,N2-dimethylguanosine	2.408	1.095–5.296	0.029	0.125
**Pyrimidine Metabolism, Uracil containing**				
pseudouridine	1.513	1.207–1.896	<0.001	0.003
3-ureidopropionate	5.255	1.602–17.240	0.006	0.039
3-(3-amino-3-carboxypropyl)uridine*	1.687	1.120–2.542	0.012	0.067
**Pyrimidine Metabolism, Thymine containing**				
thymidine	1.180	1.159–1.200	<0.001	<0.001
5,6-dihydrothymine	1.839	1.018–3.324	0.044	0.169
**Dinucleotide**				
(3'-5')-adenylylguanosine*	0.406	0.228–0.723	0.002	0.017
(3'-5')-cytidylylcytidine*	0.396	0.390–0.402	<0.001	<0.001
(3'-5')-uridylylcytidine*	0.321	0.256–0.403	<0.001	<0.001
**COFACTORS AND VITAMINS**				
**Nicotinate and Nicotinamide Metabolism**				
nicotinate	4.753	1.346–16.789	0.015	0.079
nicotinate ribonucleoside	3.863	2.237–6.672	<0.001	<0.001
nicotinamide riboside	2.980	1.281–6.931	0.011	0.063
trigonelline (N'-methylnicotinate)	3.607	1.915–6.795	<0.001	<0.001
**Riboflavin Metabolism**				
riboflavin (vitamin B2)	1.576	1.264–1.965	<0.001	<0.001
flavin adenine dinucleotide (FAD)	1.819	1.181–2.802	0.007	0.041
flavin mononucleotide (FMN)	0.601	0.383–0.942	0.027	0.118
**Ascorbate and Aldarate Metabolism**				
oxalate (ethanedioate)	0.502	0.345–0.731	<0.001	0.003
**Tocopherol Metabolism**				
gamma-CEHC glucuronide*	0.367	0.303–0.443	<0.001	<0.001
alpha-CEHC sulfate	4.877	1.287–18.471	0.020	0.095
**Tetrahydrobiopterin Metabolism**				
biopterin	2.656	2.310–3.055	<0.001	<0.001
**Hemoglobin and Porphyrin Metabolism**				
protoporphyrin IX	2.089	1.347–3.238	<0.001	0.008
heme	0.618	0.425–0.898	0.012	0.064
biliverdin	7.097	1.792–28.104	0.005	0.034

Metabolite	Fold Change (NEC/Control)	95% CI	P value	Q value
**Vitamin B6 Metabolism**				
pyridoxate	4.091	2.455–6.818	<0.001	<0.001
**XENOBIOTICS**				
**Benzoate Metabolism**				
mandelate	12.386	3.007–51.014	<0.001	0.004
guaiacol sulfate	2.510	1.377–4.574	0.003	0.020
4-methylcatechol sulfate	4.073	1.251–13.264	0.020	0.095
3-methoxycatechol sulfate (2)	9.397	4.193–21.062	<0.001	<0.001
propyl 4-hydroxybenzoate sulfate	3.003	2.623–3.439	<0.001	<0.001
p-cresol sulfate	3.521	1.418–8.747	0.007	0.041
**Xanthine Metabolism**				
theophylline	1.978	1.581–2.474	<0.001	<0.001
1-methylurate	4.862	2.229–10.606	<0.001	<0.001
**Food Component/Plant**				
vanillin	8.283	1.982–34.619	0.004	0.027
2,8-quinolinediol sulfate	3.217	1.111–9.311	0.031	0.133
3-hydroxyindolin-2-one	29.220	5.423–157.456	<0.001	<0.001
erythritol	3.274	1.708–6.279	<0.001	0.003
quinate	3.116	1.503–6.461	0.002	0.017
stachydrine	10.507	1.313–84.068	0.027	0.118
3-hydroxystachydrine*	2.268	1.431–3.595	<0.001	0.004
thymol sulfate	5.543	3.386–9.077	<0.001	<0.001
pyrraline	1.311	1.142–1.505	<0.001	0.001
2-keto-3-deoxy-gluconate	1.529	1.332–1.755	<0.001	<0.001
methyl vanillate sulfate	14.288	7.532–27.105	<0.001	<0.001
2-aminophenol	0.853	0.762–0.955	0.006	0.037
**Drug - Analgesics, Anesthetics**				
lidocaine	7.323	1.016–52.772	0.048	0.181
**Drug - Antibiotic**				
triclosan sulfate	7.887	1.364–45.591	0.021	0.100
**Drug - Gastrointestinal**				
famotidine	4.370	2.444–7.813	<0.001	<0.001
**Drug - Neurological**				
rocuronium	0.497	0.321–0.770	0.002	0.014
**PARTIALLY CHARACTERIZED MOLECULES**				
**Partially Characterized Molecules**				
GlcNAc sulfate conjugate of C21H34O2 steroid**	2.219	1.115–4.418	0.023	0.107
bilirubin degradation product, C16H18N2O5 (3)**	0.589	0.357–0.971	0.038	0.155

Supplementary Table S2. Detection frequencies of annotated metabolites with nominally significant differential abundance in NEC intestinal tissue.

Detection frequency was defined as the proportion of samples with a measured (non-missing) value before missing-value imputation. Frequencies are shown separately for control (n=8) and NEC (n=13) samples. The table includes the 255 annotated metabolites with nominal p<0.05 in the mixed-effects analysis reported in Supplementary Table S1. Difference is calculated as NEC detection percentage minus control detection percentage and is reported in percentage points (pp).

Metabolite	Control detected (n/8)	Control detection (%)	NEC detected (n/13)	NEC detection (%)	Difference (pp)
betaine aldehyde	8/8	100.0	13/13	100.0	+0.0
1-methylhistidine	8/8	100.0	13/13	100.0	+0.0
N-acetylhistidine	8/8	100.0	13/13	100.0	+0.0
imidazole propionate	8/8	100.0	13/13	100.0	+0.0
imidazole lactate	8/8	100.0	13/13	100.0	+0.0
histamine	8/8	100.0	13/13	100.0	+0.0
1-methylhistamine	8/8	100.0	12/13	92.3	-7.7
1-ribosyl-imidazoleacetate*	8/8	100.0	13/13	100.0	+0.0
N-acetylhistamine	7/8	87.5	5/13	38.5	-49.0
N2-acetyl,N6,N6-dimethyllysine	2/8	25.0	5/13	38.5	+13.5
pipecolate	8/8	100.0	13/13	100.0	+0.0
cadaverine	6/8	75.0	7/13	53.8	-21.2
N-acetylphenylalanine	8/8	100.0	13/13	100.0	+0.0
phenyllactate (PLA)	8/8	100.0	13/13	100.0	+0.0
4-hydroxyphenylacetate	2/8	25.0	9/13	69.2	+44.2
3-(4-hydroxyphenyl)lactate	8/8	100.0	13/13	100.0	+0.0
4-methoxyphenol sulfate	8/8	100.0	13/13	100.0	+0.0
vanillactate	8/8	100.0	13/13	100.0	+0.0
vanillylmandelate (VMA)	5/8	62.5	13/13	100.0	+37.5
3-methoxytyramine sulfate	8/8	100.0	13/13	100.0	+0.0
homovanillate sulfate	6/8	75.0	12/13	92.3	+17.3
tyramine O-sulfate	7/8	87.5	8/13	61.5	-26.0
vanillic alcohol sulfate	1/8	12.5	7/13	53.8	+41.3
oxindolylalanine	8/8	100.0	13/13	100.0	+0.0
tryptophan betaine	7/8	87.5	13/13	100.0	+12.5
N-acetylkynurenine (2)	5/8	62.5	12/13	92.3	+29.8
kynurenate	8/8	100.0	13/13	100.0	+0.0
8-methoxykynurenate	6/8	75.0	13/13	100.0	+25.0
5-hydroxyindoleacetate	8/8	100.0	7/13	53.8	-46.2
indolelactate	8/8	100.0	13/13	100.0	+0.0
indole-3-carboxylate	8/8	100.0	13/13	100.0	+0.0

Metabolite	Control detected (n/8)	Control detection (%)	NEC detected (n/13)	NEC detection (%)	Difference (pp)
3-indoxyl sulfate	4/8	50.0	12/13	92.3	+42.3
N-acetylleucine	8/8	100.0	13/13	100.0	+0.0
alpha-hydroxyisocaproate	5/8	62.5	13/13	100.0	+37.5
isovalerylglycine	6/8	75.0	12/13	92.3	+17.3
beta-hydroxyisovalerate	8/8	100.0	13/13	100.0	+0.0
3-methylglutaconate	8/8	100.0	13/13	100.0	+0.0
N-acetylisoleucine	8/8	100.0	13/13	100.0	+0.0
2-hydroxy-3-methylvalerate	5/8	62.5	12/13	92.3	+29.8
N-acetylvaline	8/8	100.0	13/13	100.0	+0.0
alpha-hydroxyisovalerate	8/8	100.0	13/13	100.0	+0.0
3-hydroxyisobutyrate	8/8	100.0	13/13	100.0	+0.0
2,3-dihydroxy-5-methylthio-4-pentenoate (DMTPA)*	8/8	100.0	13/13	100.0	+0.0
S-methylcysteine sulfoxide	8/8	100.0	12/13	92.3	-7.7
cysteine sulfinic acid	8/8	100.0	13/13	100.0	+0.0
N-acetyltaurine	8/8	100.0	13/13	100.0	+0.0
urea	8/8	100.0	13/13	100.0	+0.0
N-acetylproline	1/8	12.5	6/13	46.2	+33.7
N-methylproline	7/8	87.5	11/13	84.6	-2.9
N-monomethylarginine	7/8	87.5	12/13	92.3	+4.8
guanidinoacetate	8/8	100.0	13/13	100.0	+0.0
creatinine	8/8	100.0	13/13	100.0	+0.0
N-acetylputrescine	8/8	100.0	13/13	100.0	+0.0
N-acetyl-isoputreanine	8/8	100.0	13/13	100.0	+0.0
diacetylspermidine*	3/8	37.5	12/13	92.3	+54.8
(N(1) + N(8))-acetylspermidine	8/8	100.0	13/13	100.0	+0.0
spermine	8/8	100.0	13/13	100.0	+0.0
N(1)-acetylspermine	8/8	100.0	13/13	100.0	+0.0
N1,N12-diacetylspermine	8/8	100.0	13/13	100.0	+0.0
5-methylthioadenosine (MTA)	8/8	100.0	13/13	100.0	+0.0
4-acetamidobutanoate	8/8	100.0	13/13	100.0	+0.0
glutathione, oxidized (GSSG)	5/8	62.5	7/13	53.8	-8.7
2-hydroxybutyrate/2-hydroxyisobutyrate	8/8	100.0	13/13	100.0	+0.0
gamma-glutamylalanine	4/8	50.0	7/13	53.8	+3.8
gamma-glutamylhistidine	8/8	100.0	13/13	100.0	+0.0

Metabolite	Control detected (n/8)	Control detection (%)	NEC detected (n/13)	NEC detection (%)	Difference (pp)
gamma-glutamylvaline	8/8	100.0	12/13	92.3	-7.7
cyclo(leu-pro)	2/8	25.0	5/13	38.5	+13.5
glycylleucine	8/8	100.0	13/13	100.0	+0.0
leucylalanine	1/8	12.5	7/13	53.8	+41.3
lysylleucine	6/8	75.0	8/13	61.5	-13.5
threonylphenylalanine	2/8	25.0	7/13	53.8	+28.8
valylglycine	3/8	37.5	11/13	84.6	+47.1
valylleucine	8/8	100.0	13/13	100.0	+0.0
leucylglutamine*	8/8	100.0	12/13	92.3	-7.7
phenylacetylglutamine	8/8	100.0	13/13	100.0	+0.0
pyroglutamylglycine	8/8	100.0	13/13	100.0	+0.0
fructose 6-phosphate	8/8	100.0	12/13	92.3	-7.7
2,3-diphosphoglycerate	6/8	75.0	6/13	46.2	-28.8
ribose 1-phosphate	7/8	87.5	10/13	76.9	-10.6
sedoheptulose-7-phosphate	8/8	100.0	12/13	92.3	-7.7
maltose	6/8	75.0	11/13	84.6	+9.6
galactose 1-phosphate	7/8	87.5	11/13	84.6	-2.9
UDP-glucose	1/8	12.5	9/13	69.2	+56.7
UDP-galactose	1/8	12.5	6/13	46.2	+33.7
UDP-N-acetylglucosamine/galactosamine	5/8	62.5	11/13	84.6	+22.1
erythronate*	8/8	100.0	13/13	100.0	+0.0
heptanoate (7:0)	5/8	62.5	10/13	76.9	+14.4
palmitate (16:0)	8/8	100.0	13/13	100.0	+0.0
margarate (17:0)	8/8	100.0	13/13	100.0	+0.0
stearate (18:0)	8/8	100.0	13/13	100.0	+0.0
nonadecanoate (19:0)	8/8	100.0	13/13	100.0	+0.0
arachidate (20:0)	8/8	100.0	13/13	100.0	+0.0
palmitoleate (16:1n7)	8/8	100.0	13/13	100.0	+0.0
heptadecenoate (17:1)	8/8	100.0	13/13	100.0	+0.0
oleate/vaccenate (18:1)	8/8	100.0	12/13	92.3	-7.7
nonadecenoate (19:1)	8/8	100.0	12/13	92.3	-7.7
eicosenoate (20:1)	8/8	100.0	13/13	100.0	+0.0
erucate (22:1n9)	8/8	100.0	13/13	100.0	+0.0
docosatrienoate (22:3n3)	8/8	100.0	13/13	100.0	+0.0

Metabolite	Control detected (n/8)	Control detection (%)	NEC detected (n/13)	NEC detection (%)	Difference (pp)
linoleate (18:2n6)	8/8	100.0	13/13	100.0	+0.0
dihomo-linoleate (20:2n6)	8/8	100.0	13/13	100.0	+0.0
arachidonate (20:4n6)	8/8	100.0	13/13	100.0	+0.0
adrenate (22:4n6)	8/8	100.0	13/13	100.0	+0.0
docosapentaenoate (n6 DPA; 22:5n6)	8/8	100.0	12/13	92.3	-7.7
docosadienoate (22:2n6)	8/8	100.0	13/13	100.0	+0.0
(16 or 17)-methylstearate (a19:0 or i19:0)	8/8	100.0	12/13	92.3	-7.7
2-hydroxyadipate	1/8	12.5	8/13	61.5	+49.0
tetradecanedioate (C14-DC)	8/8	100.0	13/13	100.0	+0.0
hexadecanedioate (C16-DC)	8/8	100.0	13/13	100.0	+0.0
hexadecenedioate (C16:1-DC)*	5/8	62.5	6/13	46.2	-16.3
N-acetyl-2-aminooctanoate*	8/8	100.0	13/13	100.0	+0.0
valerylglycine	7/8	87.5	11/13	84.6	-2.9
myristoylcarnitine (C14)	8/8	100.0	13/13	100.0	+0.0
palmitoleoylcarnitine (C16:1)*	8/8	100.0	13/13	100.0	+0.0
octadecenedioylcarnitine (C18:1-DC)*	4/8	50.0	8/13	61.5	+11.5
3-hydroxypalmitoylcarnitine	8/8	100.0	13/13	100.0	+0.0
2-hydroxyarachidate*	8/8	100.0	12/13	92.3	-7.7
2-hydroxynervonate*	8/8	100.0	13/13	100.0	+0.0
3-hydroxyoctanoate	8/8	100.0	13/13	100.0	+0.0
3-hydroxydecanoate	8/8	100.0	13/13	100.0	+0.0
12,13-DiHOME	6/8	75.0	12/13	92.3	+17.3
2,4-dihydroxybutyrate	8/8	100.0	11/13	84.6	-15.4
5-HETE	7/8	87.5	12/13	92.3	+4.8
N-oleoyltaurine	8/8	100.0	13/13	100.0	+0.0
N-stearoyltaurine	8/8	100.0	13/13	100.0	+0.0
linolenoyl ethanolamide (18:3)*	2/8	25.0	6/13	46.2	+21.2
N-oleoylserine	7/8	87.5	10/13	76.9	-10.6
glycerophosphoethanolamine	8/8	100.0	13/13	100.0	+0.0
1-palmitoyl-2-pentadecanoyl-GPC (16:0/15:0)*	8/8	100.0	13/13	100.0	+0.0
1-palmitoyl-2-linoleoyl-GPC (16:0/18:2)	8/8	100.0	13/13	100.0	+0.0
1-palmitoyl-2-eicosapentaenoyl-GPC (16:0/20:5)*	8/8	100.0	12/13	92.3	-7.7
1-palmitoyl-2-docosahexaenoyl-GPC (16:0/22:6)	8/8	100.0	12/13	92.3	-7.7
1-stearoyl-2-linoleoyl-GPC (18:0/18:2)*	8/8	100.0	13/13	100.0	+0.0

Metabolite	Control detected (n/8)	Control detection (%)	NEC detected (n/13)	NEC detection (%)	Difference (pp)
1,2-dioleoyl-GPC (18:1/18:1)	5/8	62.5	10/13	76.9	+14.4
1,2-dilinoleoyl-GPC (18:2/18:2)	8/8	100.0	13/13	100.0	+0.0
1-palmitoyl-2-linoleoyl-GPE (16:0/18:2)	8/8	100.0	11/13	84.6	-15.4
1-palmitoyl-2-eicosapentaenoyl-GPE (16:0/20:5)*	8/8	100.0	9/13	69.2	-30.8
1-stearoyl-2-oleoyl-GPE (18:0/18:1)	8/8	100.0	12/13	92.3	-7.7
1-stearoyl-2-dihomo-linolenoyl-GPE (18:0/20:3n3 or n6)*	8/8	100.0	11/13	84.6	-15.4
1-stearoyl-2-docosahexaenoyl-GPE (18:0/22:6)*	8/8	100.0	11/13	84.6	-15.4
1-stearoyl-2-oleoyl-GPS (18:0/18:1)	8/8	100.0	13/13	100.0	+0.0
1-stearoyl-2-docosahexaenoyl-GPS (18:0/22:6)*	8/8	100.0	13/13	100.0	+0.0
1-palmitoleoyl-GPC (16:1)*	8/8	100.0	13/13	100.0	+0.0
1-palmitoyl-GPE (16:0)	8/8	100.0	13/13	100.0	+0.0
2-stearoyl-GPE (18:0)*	8/8	100.0	13/13	100.0	+0.0
2-arachidonoyl-GPE (20:4)*	8/8	100.0	10/13	76.9	-23.1
1-palmitoyl-GPS (16:0)*	8/8	100.0	13/13	100.0	+0.0
1-oleoyl-GPS (18:1)	8/8	100.0	13/13	100.0	+0.0
1-oleoyl-GPG (18:1)*	8/8	100.0	13/13	100.0	+0.0
1-oleoyl-GPI (18:1)	8/8	100.0	13/13	100.0	+0.0
1-arachidonoyl-GPI (20:4)*	8/8	100.0	13/13	100.0	+0.0
1-(1-enyl-palmitoyl)-2-oleoyl-GPE (P-16:0/18:1)*	8/8	100.0	13/13	100.0	+0.0
1-(1-enyl-palmitoyl)-2-linoleoyl-GPE (P-16:0/18:2)*	8/8	100.0	13/13	100.0	+0.0
1-(1-enyl-stearoyl)-2-oleoyl-GPE (P-18:0/18:1)	8/8	100.0	12/13	92.3	-7.7
1-(1-enyl-palmitoyl)-2-arachidonoyl-GPC (P-16:0/20:4)*	8/8	100.0	13/13	100.0	+0.0
1-palmityl-GPE (O-16:0)*	8/8	100.0	13/13	100.0	+0.0
1-stearyl-GPE (O-18:0)*	8/8	100.0	13/13	100.0	+0.0
1-(1-enyl-stearoyl)-GPE (P-18:0)*	8/8	100.0	13/13	100.0	+0.0
1-myristoylglycerol (14:0)	8/8	100.0	12/13	92.3	-7.7
1-palmitoylglycerol (16:0)	8/8	100.0	13/13	100.0	+0.0
1-palmitoleoylglycerol (16:1)*	8/8	100.0	13/13	100.0	+0.0
1-linoleoylglycerol (18:2)	8/8	100.0	13/13	100.0	+0.0
1-arachidonylglycerol (20:4)	8/8	100.0	13/13	100.0	+0.0
1-docosahexaenoylglycerol (22:6)	8/8	100.0	13/13	100.0	+0.0
2-palmitoylglycerol (16:0)	7/8	87.5	12/13	92.3	+4.8
2-palmitoleoylglycerol (16:1)*	7/8	87.5	12/13	92.3	+4.8
2-oleoylglycerol (18:1)	8/8	100.0	13/13	100.0	+0.0

Metabolite	Control detected (n/8)	Control detection (%)	NEC detected (n/13)	NEC detection (%)	Difference (pp)
2-linoleoylglycerol (18:2)	8/8	100.0	13/13	100.0	+0.0
palmitoyl-linolenoyl-glycerol (16:0/18:3) [2]*	4/8	50.0	7/13	53.8	+3.8
palmitoyl-arachidonoyl-glycerol (16:0/20:4) [2]*	8/8	100.0	13/13	100.0	+0.0
oleoyl-linoleoyl-glycerol (18:1/18:2) [1]	7/8	87.5	11/13	84.6	-2.9
linoleoyl-linoleoyl-glycerol (18:2/18:2) [1]*	6/8	75.0	10/13	76.9	+1.9
oleoyl-arachidonoyl-glycerol (18:1/20:4) [1]*	8/8	100.0	12/13	92.3	-7.7
galactosylglycerol	5/8	62.5	11/13	84.6	+22.1
sphingadienine	8/8	100.0	13/13	100.0	+0.0
N-stearoyl-sphinganine (d18:0/18:0)*	8/8	100.0	12/13	92.3	-7.7
glycosyl-N-stearoyl-sphingosine (d18:1/18:0)	3/8	37.5	10/13	76.9	+39.4
sphingomyelin (d18:0/20:0, d16:0/22:0)*	8/8	100.0	11/13	84.6	-15.4
3-hydroxy-3-methylglutarate	8/8	100.0	13/13	100.0	+0.0
mevalonate	8/8	100.0	13/13	100.0	+0.0
4-cholesten-3-one	8/8	100.0	13/13	100.0	+0.0
7-hydroxycholesterol (alpha or beta)	5/8	62.5	9/13	69.2	+6.7
pregnenolone sulfate	8/8	100.0	13/13	100.0	+0.0
17alpha-hydroxypregnenolone 3-sulfate	7/8	87.5	13/13	100.0	+12.5
21-hydroxypregnenolone disulfate	8/8	100.0	13/13	100.0	+0.0
pregnenediol sulfate (C21H34O5S)*	8/8	100.0	13/13	100.0	+0.0
pregnenediol disulfate (C21H34O8S2)*	8/8	100.0	13/13	100.0	+0.0
pregnenetriol sulfate*	8/8	100.0	13/13	100.0	+0.0
pregnenetriol disulfate*	8/8	100.0	13/13	100.0	+0.0
5alpha-pregnan-3beta,20alpha-diol disulfate	3/8	37.5	13/13	100.0	+62.5
cortisol	5/8	62.5	11/13	84.6	+22.1
cortisol 21-sulfate	3/8	37.5	11/13	84.6	+47.1
cortisone	7/8	87.5	13/13	100.0	+12.5
dehydroepiandrosterone sulfate (DHEA-S)	8/8	100.0	13/13	100.0	+0.0
16alpha-hydroxy DHEA 3-sulfate	8/8	100.0	13/13	100.0	+0.0
androstenediol (3beta,17beta) disulfate (1)	7/8	87.5	13/13	100.0	+12.5
androstenediol (3beta,17beta) disulfate (2)	8/8	100.0	13/13	100.0	+0.0
andro steroid monosulfate C19H28O6S (1)*	8/8	100.0	13/13	100.0	+0.0
tauro-alpha-muricholate	7/8	87.5	13/13	100.0	+12.5
glycoursodeoxycholate	3/8	37.5	4/13	30.8	-6.7
taurohyodeoxycholic acid	3/8	37.5	9/13	69.2	+31.7

Metabolite	Control detected (n/8)	Control detection (%)	NEC detected (n/13)	NEC detection (%)	Difference (pp)
glycocholenate sulfate*	8/8	100.0	12/13	92.3	-7.7
N6-carbamoylthreonyladenosine	8/8	100.0	13/13	100.0	+0.0
2'-deoxyadenosine	3/8	37.5	7/13	53.8	+16.3
7-methylguanine	8/8	100.0	13/13	100.0	+0.0
N2,N2-dimethylguanosine	8/8	100.0	13/13	100.0	+0.0
pseudouridine	8/8	100.0	13/13	100.0	+0.0
3-ureidopropionate	8/8	100.0	13/13	100.0	+0.0
3-(3-amino-3-carboxypropyl)uridine*	8/8	100.0	13/13	100.0	+0.0
thymidine	8/8	100.0	12/13	92.3	-7.7
5,6-dihydrothymine	7/8	87.5	12/13	92.3	+4.8
(3'-5')-adenylylguanosine*	8/8	100.0	12/13	92.3	-7.7
(3'-5')-cytidylylcytidine*	7/8	87.5	11/13	84.6	-2.9
(3'-5')-uridylylcytidine*	7/8	87.5	10/13	76.9	-10.6
nicotinate	3/8	37.5	9/13	69.2	+31.7
nicotinate ribonucleoside	4/8	50.0	9/13	69.2	+19.2
nicotinamide riboside	7/8	87.5	12/13	92.3	+4.8
trigonelline (N'-methylnicotinate)	8/8	100.0	13/13	100.0	+0.0
riboflavin (vitamin B2)	7/8	87.5	13/13	100.0	+12.5
flavin adenine dinucleotide (FAD)	8/8	100.0	12/13	92.3	-7.7
flavin mononucleotide (FMN)	8/8	100.0	13/13	100.0	+0.0
oxalate (ethanedioate)	8/8	100.0	13/13	100.0	+0.0
gamma-CEHC glucuronide*	3/8	37.5	6/13	46.2	+8.7
alpha-CEHC sulfate	7/8	87.5	9/13	69.2	-18.3
biopterin	7/8	87.5	12/13	92.3	+4.8
protoporphyrin IX	3/8	37.5	3/13	23.1	-14.4
heme	8/8	100.0	13/13	100.0	+0.0
biliverdin	8/8	100.0	13/13	100.0	+0.0
pyridoxate	8/8	100.0	13/13	100.0	+0.0
mandelate	6/8	75.0	13/13	100.0	+25.0
guaiacol sulfate	8/8	100.0	13/13	100.0	+0.0
4-methylcatechol sulfate	6/8	75.0	12/13	92.3	+17.3
3-methoxycatechol sulfate (2)	3/8	37.5	13/13	100.0	+62.5
propyl 4-hydroxybenzoate sulfate	7/8	87.5	11/13	84.6	-2.9
p-cresol sulfate	8/8	100.0	13/13	100.0	+0.0

Metabolite	Control detected (n/8)	Control detection (%)	NEC detected (n/13)	NEC detection (%)	Difference (pp)
theophylline	2/8	25.0	11/13	84.6	+59.6
1-methylurate	7/8	87.5	12/13	92.3	+4.8
vanillin	7/8	87.5	10/13	76.9	-10.6
2,8-quinolinediol sulfate	1/8	12.5	3/13	23.1	+10.6
3-hydroxyindolin-2-one	2/8	25.0	7/13	53.8	+28.8
erythritol	8/8	100.0	13/13	100.0	+0.0
quinate	8/8	100.0	11/13	84.6	-15.4
stachydrine	8/8	100.0	13/13	100.0	+0.0
3-hydroxystachydrine*	2/8	25.0	12/13	92.3	+67.3
thymol sulfate	2/8	25.0	12/13	92.3	+67.3
pyrraline	3/8	37.5	5/13	38.5	+1.0
2-keto-3-deoxy-gluconate	8/8	100.0	13/13	100.0	+0.0
methyl vanillate sulfate	2/8	25.0	11/13	84.6	+59.6
2-aminophenol	5/8	62.5	12/13	92.3	+29.8
lidocaine	1/8	12.5	6/13	46.2	+33.7
triclosan sulfate	4/8	50.0	10/13	76.9	+26.9
famotidine	3/8	37.5	3/13	23.1	-14.4
rocuronium	2/8	25.0	5/13	38.5	+13.5
GlcNAc sulfate conjugate of C21H34O2 steroid**	8/8	100.0	12/13	92.3	-7.7
bilirubin degradation product, C16H18N2O5 (3)**	4/8	50.0	12/13	92.3	+42.3
